# Ketamine and its metabolite, (2R,6R)-HNK, restore hippocampal LTP and long-term spatial memory in the Wistar-Kyoto rat model of depression

**DOI:** 10.1186/s13041-020-00627-z

**Published:** 2020-06-16

**Authors:** Lily R. Aleksandrova, Yu Tian Wang, Anthony G. Phillips

**Affiliations:** 1 Djavad Mowafaghian Centre for Brain Health, University of British Columbia, Vancouver, BC Canada; 2 Department of Psychiatry, University of British Columbia, Vancouver, BC Canada; 3 Department of Medicine, University of British Columbia, Vancouver, BC Canada

**Keywords:** Ketamine, Synaptic plasticity, LTP, Hippocampus, HNK, Antidepressant, Memory and cognition, Wistar-Kyoto rat, Model of depression

## Abstract

Accumulating evidence implicates dysregulation of hippocampal synaptic plasticity in the pathophysiology of depression. However, the effects of ketamine on synaptic plasticity and their contribution to its mechanism of action as an antidepressant, are still unclear. We investigated ketamine’s effects on in vivo dorsal hippocampal (dHPC) synaptic plasticity and their role in mediating aspects of antidepressant activity in the Wistar-Kyoto (WKY) model of depression. dHPC long-term potentiation (LTP) was significantly impaired in WKY rats compared to Wistar controls. Importantly, a single low dose (5 mg/kg, ip) of ketamine or its metabolite, (2R,6R)-HNK, rescued the LTP deficit in WKY rats at 3.5 h but not 30 min following injection, with residual effects at 24 h, indicating a delayed, sustained facilitatory effect on dHPC synaptic plasticity. Consistent with the observed dHPC LTP deficit, WKY rats exhibited impaired hippocampal-dependent long-term spatial memory as measured by the novel object location recognition test (NOLRT), which was effectively restored by pre-treatment with both ketamine or (2R,6R)-HNK. In contrast, in WKYs, which display abnormal stress coping, ketamine, but not (2R,6R)-HNK, had rapid and sustained effects in the forced swim test (FST), a commonly used preclinical screen for antidepressant-like activity. The differential effects of (2R,6R)-HNK observed here reveal a dissociation between drug effects on FST immobility and dHPC synaptic plasticity. Therefore, in the WKY rat model, restoring dHPC LTP was not correlated with ketamine’s effects in FST, but importantly, may have contributed to the reversal of hippocampal-dependent cognitive deficits, which are critical features of clinical depression. Our findings support the theory that ketamine may reverse the stress-induced loss of connectivity in key neural circuits by engaging synaptic plasticity processes to “reset the system”.

## Introduction

Accumulating evidence implicates dysfunction within glutamatergic systems and dysregulation of synaptic plasticity in the pathophysiology of depression [1–7]. Preclinical studies have established that chronic stress is associated with pathological glutamate excitotoxicity and synaptic dysfunction, leading to reductions in dendritic branching and spine density, and eventually neuronal atrophy of pyramidal neurons in areas implicated in major depressive disorder (MDD), particularly the hippocampus (HPC) and prefrontal cortex (PFC) [1, 3, 5, 8–10]. Importantly, electrophysiological studies indicate that stress can perturb the normal balance in synaptic plasticity, by inhibiting long-term potentiation (LTP) and/or facilitating long-term depression (LTD), mainly in the rodent HPC (for review, [7]). When prolonged (e.g. under chronic stress), such an imbalance may predispose toward synaptic destabilization and neuronal atrophy, possibly mediating the structural and functional findings in MDD [3, 7, 11–14]. Importantly, depressed patients show significant grey matter volume reductions, particularly in the HPC and PFC, as well as various cognitive deficits (e.g. in attention, episodic memory and executive function) [3, 7, 11–14]. These effects, along with the core symptoms of emotional dysregulation and anhedonia [15–17], could all be mediated by impaired synaptic plasticity and loss of connectivity between key brain regions highly vulnerable to stress (for review, [7]). Despite recent progress, further research is clearly needed to clarify the role of synaptic plasticity in the pathophysiology of depression and in mediating antidepressant response.

Ketamine’s efficacy in treatment-resistant depression (TRD, i.e. in patients who have previously failed to respond to two or more classical antidepressants), holds promise for a new generation of much needed, superior antidepressant agents. Given its short plasma half-life (~ 1–3 h), ketamine’s sustained antidepressant actions (~days-weeks) appear to be due to activation of crucial downstream signaling cascades as a secondary consequence of inhibiting the glutamate N-methyl-D-aspartate receptor (NMDAR), resulting in long-lasting adaptations in key neural circuits [3, 18–20]. Ketamine recruits intracellular signaling cascades, particularly those involving the brain-derived neurotrophic factor (BDNF) and the mammalian target of rapamycin (mTOR), and is thought to initiate an LTP-like process involving acute activation of the glutamate α-amino-3-hydroxy-5-methyl-4-isoxazolepropionic acid receptor (AMPAR) and sustained enhancement of AMPAR-mediated transmission [18]. Ultimately, ketamine increases the ratio of AMPAR to NMDAR throughput via directly blocking NMDARs and indirectly enhancing AMPAR function, leading to synaptic protein synthesis, synaptogenesis and reversal of stress-induced synaptic dysfunction and neuronal atrophy in brain areas implicated in MDD (HPC, PFC) (for review, [7]). Although these key molecular and structural effects are now well established, mechanisms underlying the drug’s antidepressant effects on a “systems level” remain unclear. One theory is that ketamine may reverse the loss of normal connectivity between the HPC, PFC and associated regions by engaging synaptic plasticity and synaptogenesis to “reset the system” [7]. However, the number of systematic studies of ketamine’s effects on regional synaptic plasticity, especially in vivo and in the context of depression, remain limited.

In 2016, an active ketamine metabolite, (2R,6R)-hydroxynorketamine (HNK), was reported to exhibit antidepressant efficacy in rodents without NMDAR binding properties or key side effects of its parent compound [21]. These intriguing findings prompted a re-evaluation of the NMDAR hypothesis of ketamine; however, several groups subsequently failed to demonstrate any antidepressant activity of (2R,6R)-HNK in various rodent models, giving rise to controversy [22–25]. Overall, this metabolite may recapitulate some aspects of ketamine action by indirectly facilitating AMPAR-mediated transmission [19, 21, 26]; however, its exact mechanism of action and contribution to ketamine’s therapeutic effects remain unknown and warrant further investigation.

It is becoming increasingly clear that purely stress-based animal models of depression largely ignore important clinical factors, such as depression vulnerability and antidepressant resistance [27–30]. Thus, drugs that simply reverse the neurotoxic effects of stress in the otherwise normal brain may have limited efficacy and scope of antidepressant action [28, 29]. As we seek to ensure that the next generation of antidepressants will be effective for vulnerable and treatment-resistant populations, animal models should encompass not only stress-induced phenotypic parallels to clinical depression, but also aspects of heightened stress susceptibility and resistance to conventional antidepressant drugs [27, 29, 30]. Accumulating research supports the use of the Wistar-Kyoto (WKY) rat as a valid model of endogenous stress susceptibility and depression that exhibits various depressive-like phenotypes (e.g. behavioural inhibition, psychomotor slowing, anhedonia, social withdrawal, anxiety, cognitive deficits), as well as key neurochemical and endocrine parallels to MDD (e.g. deficient monoamine and neurotrophin signaling, aberrant glutamatergic function, hippocampal and cortical volume loss, and hyperactive hypothalamic-pituitary-adrenal (HPA) axis) (for review, [7]). Importantly, this strain is a model of treatment resistance to classical antidepressants (e.g. selective serotonin reuptake inhibitors, SSRIs), and responds well to novel rapid-acting antidepressant therapies proven effective in TRD, particularly ketamine [7, 29].

To evaluate the role of dorsal hippocampal (dHPC) synaptic plasticity in depression and ketamine’s antidepressant response, we used the WKY model to investigate the effects of ketamine on Schaffer collateral (SC) - CA1 LTP and their contribution to its antidepressant activity, particularly as it relates to an aspect of spatial memory function known to be mediated by this region of the hippocampus.

## Methods and materials

### Subjects

Stress-susceptible Wistar-Kyoto (WKY) and control Wistar (WIS) rats (male, age 10–12 weeks at arrival; Charles River, USA) were pair-housed under a reverse light/dark cycle, with food and water available ad libitum. Animal experiments were carried out in accordance with the Canadian Council of Animal Care and with the approval of the Animal Care Committee at the University of British Columbia.

### Drugs

Ketamine and its metabolite (2R,6R)-HNK (5 mg/kg) were dissolved in saline and administered intraperitoneally (ip) at a final volume of 1 ml/kg. Ketamine HCl was purchased from Medisca Pharmaceuticals Inc. (St-Laurent, Quebec). (2R,6R)-HNK was synthesized and verified in our lab, according to a published protocol [21] (Supplementary Methods and Fig. S1).

### Behavioural assays

After a week of acclimatization, naïve or drug-treated (WKY or WIS) rats underwent behavioural testing on three different preclinical tests (one per cohort): the open field test (OFT, 10 min session), the forced swim test (FST, 2-day procedure) or the novel object location recognition task (NOLRT, at 1 h or 24 h delay for short- and long-term memory), with drugs administered at different time points before testing. For detailed descriptions of behavioural protocols, see Supplementary Methods.

### In vivo electrophysiology

Dorsal hippocampal synaptic plasticity was evaluated using single unit field electrophysiological recordings in anesthetized rats (urethane, 1.5 g/kg, ip), according to a previously published protocol [9] (Supplementary Methods). Briefly, a stimulating and recording electrode were lowered into the SC and CA1 stratum radiatum, respectively. Evoked field excitatory post-synaptic potentials (fEPSPs) were recorded in naïve and drug-treated rats. Three stimulation protocols were applied (one per cohort) to induce weak LTP (wLTP 1x100Hz, 1 s), strong LTP (sLTP 4x100Hz, 5 min apart), or LTD (3 Hz, 900 pulses, 5 min), with drugs given at 30 min, 3.5 h or 24 h before LTP/LTD induction. Normalized fEPSP slope was analyzed and compared between experimental groups at different time points of the recording (in 5 min time bins). For detailed descriptions of electrophysiological protocols and analyses, see Supplementary Methods.

### Data analysis

Data are presented throughout as mean ± SEM, where n is the number of rats. Throughout the study, most comparisons were conducted by 1- or 2- way analysis of variance testing (ANOVA, independent groups or repeated measures, as specified in Supplementary Results) with appropriate post-hoc tests (Tukey’s or Sidak’s, as detailed in the text), or on a few occasions by a two-tailed t test. Results were analyzed and graphed using Prism 6.0 (GraphPad, San Diego, California, USA). Significance in all analyses was set at α = 0.05, with multiplicity adjusted *p* values for each comparison reported throughout. For key raw data and detailed descriptions of statistical analyses, see Supplementary Results.

## Results

### Ketamine decreases FST immobility in WKY and WIS rats

**Fig. 1:**
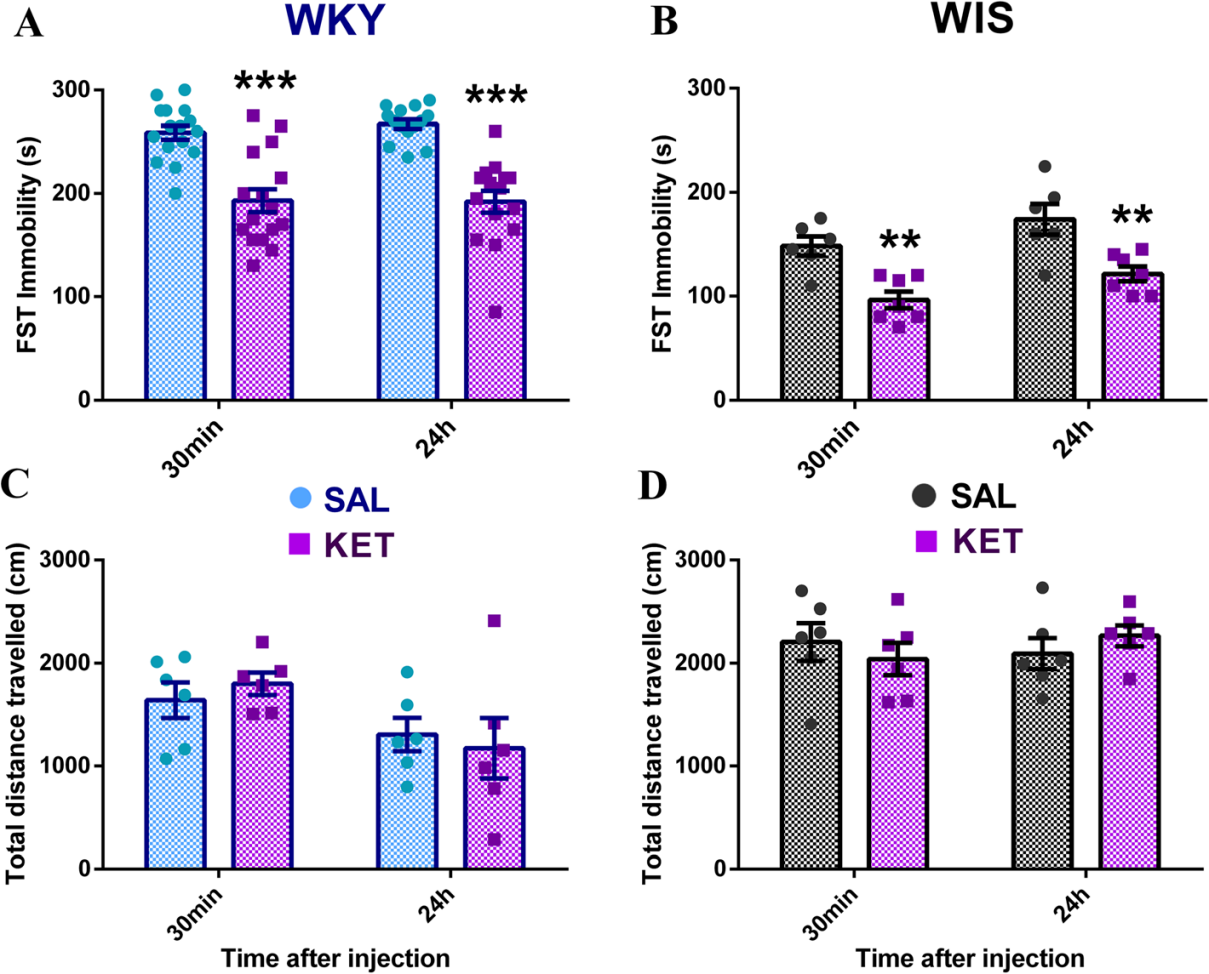
Ketamine decreases FST immobility in WKY and WIS rats without affecting locomotor activity. (A, B) Average day2 FST immobility for saline/ketamine treated WKY (*n* = 14–16/group) and WIS (*n* = 6–7/group) rats. Stress-susceptible WKYs exhibited dramatic baseline immobility compared to WIS controls (*p* < 0.0001). **a** Ketamine significantly decreased FST immobility in WKY rats at 30 min and 24 h after injection compared to saline (***p < 0.0001), with effects being comparable at 30 min and 24 h. **b** Ketamine also significantly decreased FST immobility in WIS rats at 30 min and 24 h after injection compared to saline (**p < 0.0073 and **p = 0.0063), with effects being comparable at 30 min and 24 h. **c**, **d** Average total distance travelled in the OFT for saline and ketamine treated WKY and WIS rats (*n* = 6/group). WKY rats exhibited less general locomotor activity compared to WIS controls (*p <* 0.0001). Ketamine had no effects on general locomotor activity at 30 min or 24 h post-injection in (**c**) WKY or (**d**) WIS rats. * vs. SAL; *p ≤ 0.05, **p ≤ 0.01, ***p ≤ 0.001

Consistent with the literature, stress-susceptible WKY rats were characterized by dramatic day2 FST immobility compared to normal WIS controls (30 min SAL WKY vs. WIS, two-tailed t test, *p* < 0.0001) (Fig. 1a, b), indicative of abnormal stress coping. Consistent with their behavioural inhibition phenotype, WKYs also exhibited less general locomotor activity compared to WIS rats (30 min SAL WKY vs. WIS, two-tailed t test, *p* < 0.0001) (Fig. 1c, d). Ketamine (5 mg/kg, ip) significantly decreased day2 FST immobility in both strains compared to their corresponding saline-treated controls at 30 min and 24 h after injection (significant ANOVA main effect of drug treatment, *p* < 0.0001; KET vs. SAL at 30 min/24 h, WKY: Sidak’s *p* < 0.0001 and WIS: *p* < 0.0073), with drug effects being comparable at 30 min and 24 h (no significant ANOVA time main effect or interaction, *p* > 0.05, n.s.) (Fig. 1a, b). On the other hand, ketamine had no significant effect on general locomotor activity in the OFT at these time points in either strain (no significant ANOVA drug treatment main effect or interaction, *p* > 0.05, n.s.) (Fig. 1c, d). Therefore, ketamine was found to have significant rapid (30 min) and sustained (24 h) antidepressant-like effects as indicated by the FST, in both WKY and WIS rats.

### Stress-prone WKY rats have a significant SC-CA1 LTP deficit

**Fig. 2:**
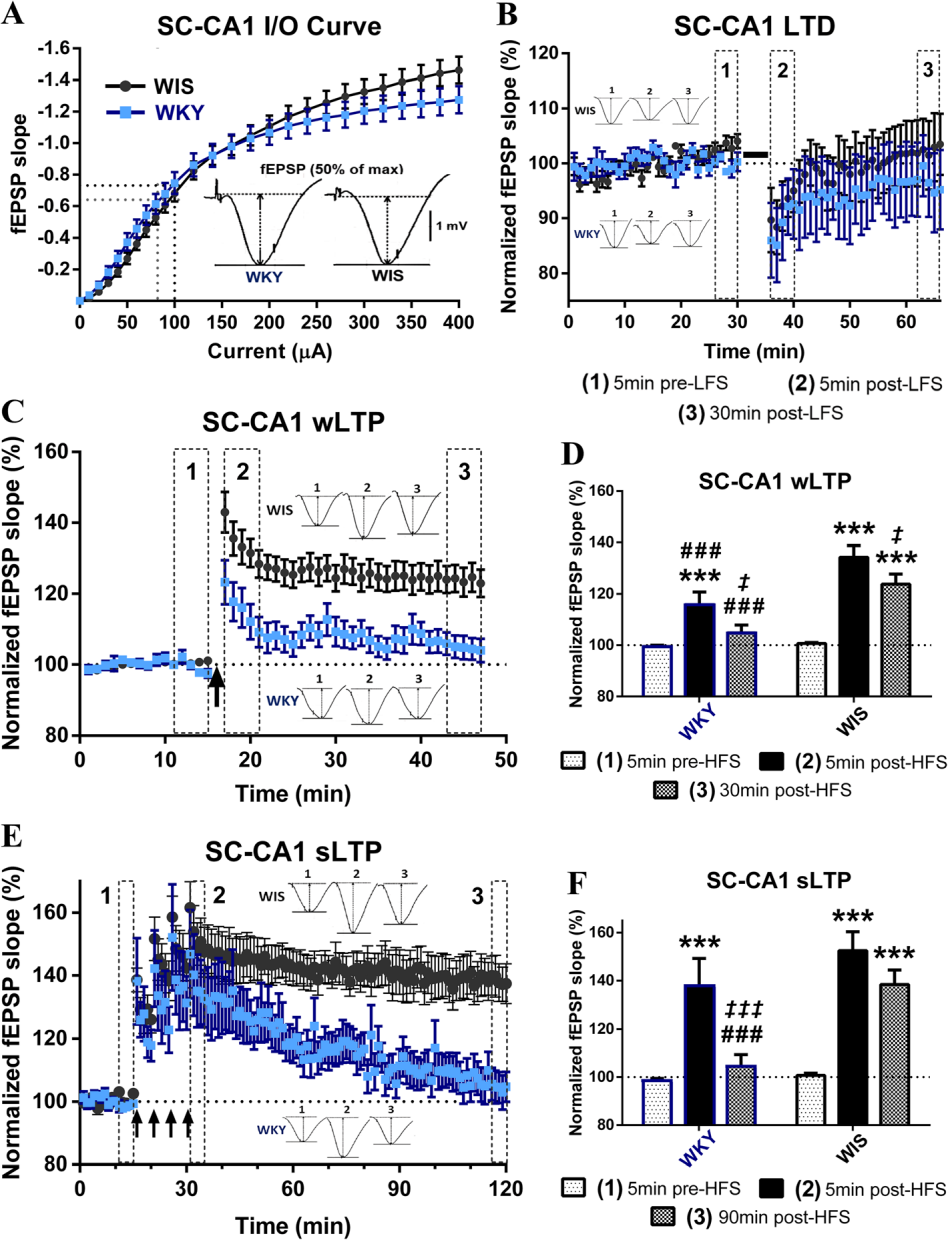
WKYs exhibit a significant SC-CA1 LTP deficit, with normal basal synaptic transmission and LTD. **a** Representative fEPSP signals and average input-output (I/O) curves of fEPSP slope for WKY and WIS rats (*n* = 19–21 rats × 2 hemispheres /strain). SC-CA1 basal synaptic transmission was comparable between the two strains. **b** LTD recordings following LFS (3 Hz, 900pulses, 5 min (solid black line)) by strain (WKY *n* = 9, WIS: *n* = 14). Although fEPSP slope was transiently reduced 5 min post-LFS in WKY and WIS rats (*p* = 0.14, n.s. and *p =* 0.04), it effectively returned to baseline within 30 min in both strains. **c**, **d** Weak LTP recordings following HSF (1 × 100 Hz, 1 s (single black arrow)) by (**c**) strain and (**d**) time bin (WKY *n* = 21, WIS: *n* = 26). While there was a significant increase in fEPSP slope 5 min post-HFS in both strains (***p = 0.0006 and ***p < 0.0001), wLTP induction was significantly compromised in WKY compared to WIS rats (# # #*p* = 0.0007), and despite some decay in both strains (‡*p =* 0.03 and ‡*p =* 0.02), significant wLTP was still observed 30 min later in control WIS (***p < 0.0001) but not WKY rats (*p* = 0.41, n.s.; vs. WIS # # #*p* = 0.0005). **e**, **f** Saturated LTP recordings following HSF (4 × 100 Hz, 1 s, 5 min apart (4 black arrows)) by (**e**) strain and (**f**) time bin (WKY *n* = 12, WIS: *n* = 14). Although fEPSP slope increased immediately post-HFS in both strains (***p < 0.0001), significant sLTP was still observed 90 min later in control WIS (***p < 0.0001) but not WKY rats (*p* = 0.76, n.s.; due to significant sLTP decay *‡ ‡ ‡p* = 0.0006; vs. WIS # # #*p* = 0.001). * vs. [1] = potentiation effect, *‡* vs. [2] = decay effect, # vs. WIS = strain effect; *p ≤ 0.05, **p ≤ 0.01, ***p ≤ 0.001

We performed in vivo extracellular recordings in anesthetized rats and found that basal SC-CA1 synaptic transmission was comparable between stress-prone WKY and control WIS rats, as indicated by similar fEPSP slope input-output curves (significant ANOVA stimulation current main effect and stimulation current x strain interaction, *p* < 0.0001, but no strain main effect; WKY vs. WIS at any current intensity, Sidak’s *p* > 0.66) (Fig. 2a). Following a low-frequency stimulation protocol (3 Hz, 900 pulses, 5 min), average fEPSP slope was transiently reduced in all rats (significant ANOVA main effect of time only, *p* = 0.0076; 5 min pre- vs. 5 min post- LFS, WKY: Tukey’s *p* = 0.14, n.s. and WIS: *p =* 0.04); however, we failed to detect any robust or long-lasting LTD in either strain (5 min pre- vs. 30 min post- LFS, Tukey’s *p >* 0.83, n.s.; 30 min post-LFS WKY vs. WIS, Sidak’s *p =* 0.77, n.s.) (Fig. 2b), indicating no significant facilitation of LTD in stress-prone WKY rats.

On the other hand, we found that SC-CA1 LTP was significantly impaired in WKYs compared to WIS controls (Fig. 2c-f). Both the induction and maintenance phases of weak LTP (wLTP) following a single train of high-frequency stimulation (HFS: 100 Hz, 1 s) were significantly less pronounced in WKY compared to WIS rats (significant time x strain ANOVA, *p* < 0.0022, 5 min post-HFS WKY vs. WIS, Sidak’s *p* = 0.0007; 30 min post-HFS WKY vs. WIS, Sidak’s *p* = 0.0005). Despite some decay in both strains (5 min post- vs. 30 min post- HFS, WKY: Tukey’s *p =* 0.03 and WIS: *p =* 0.02), significant wLTP was still observed 30 min later in control WIS rats only (5 min pre- vs. 30 min post- HFS, WKY: Tukey’s *p* = 0.41, n.s. and WIS: *p* < 0.0001) (Fig. 2c, d). At the end of the recording, 13/26 (50%) of WIS and 2/21 (9.5%) of WKY rats still expressed robust (20% or more) wLTP.

Following a stronger HFS protocol (4 trains of 100 Hz, 5 min apart), the induction phase of strong LTP (sLTP) was no longer significantly different between the two strains (significant time x strain ANOVA, *p* < 0.025; 5 min pre- vs. 5 min post- HFS, Tukey’s *p* < 0.0001 for WIS and WKY rats; 5 min post-HFS WKY vs. WIS, Sidak’s *p* = 0.30, n.s.) (Fig. 2e, f). Importantly, however, in control WIS rats, there was no significant decay in potentiation (90 min post- vs. 5 min post- HFS Tukey’s *p* = 0.17, n.s.) and significant sLTP was still observed 90 min after induction (90 min post- vs. 5 min pre- HFS Tukey’s *p* < 0.0001), whereas synaptic potentiation in the WKY strain was completely lost within 60-90 min (90 min post- vs. 5 min pre-HFS Tukey’s *p* = 0.76, n.s.; vs. 5 min post- HFS Tukey’s *p* = 0.0006; 90 min post-HFS WKY vs. WIS, Sidak’s *p* = 0.001) (Fig. 2e, f). At the end of the recording, 10/14 (71%) of WIS and 2/12 (17%) of WKY rats still expressed robust sLTP. Therefore, while the magnitude and duration of LTP were enhanced in both strains by using a stronger induction protocol to convert weak to strong LTP, we found strong evidence of a significant deficit in SC-CA1 sLTP maintenance in stress-prone WKY rats.

### Ketamine acutely restores normal SC-CA1 sLTP in WKY rats

**Fig. 3:**
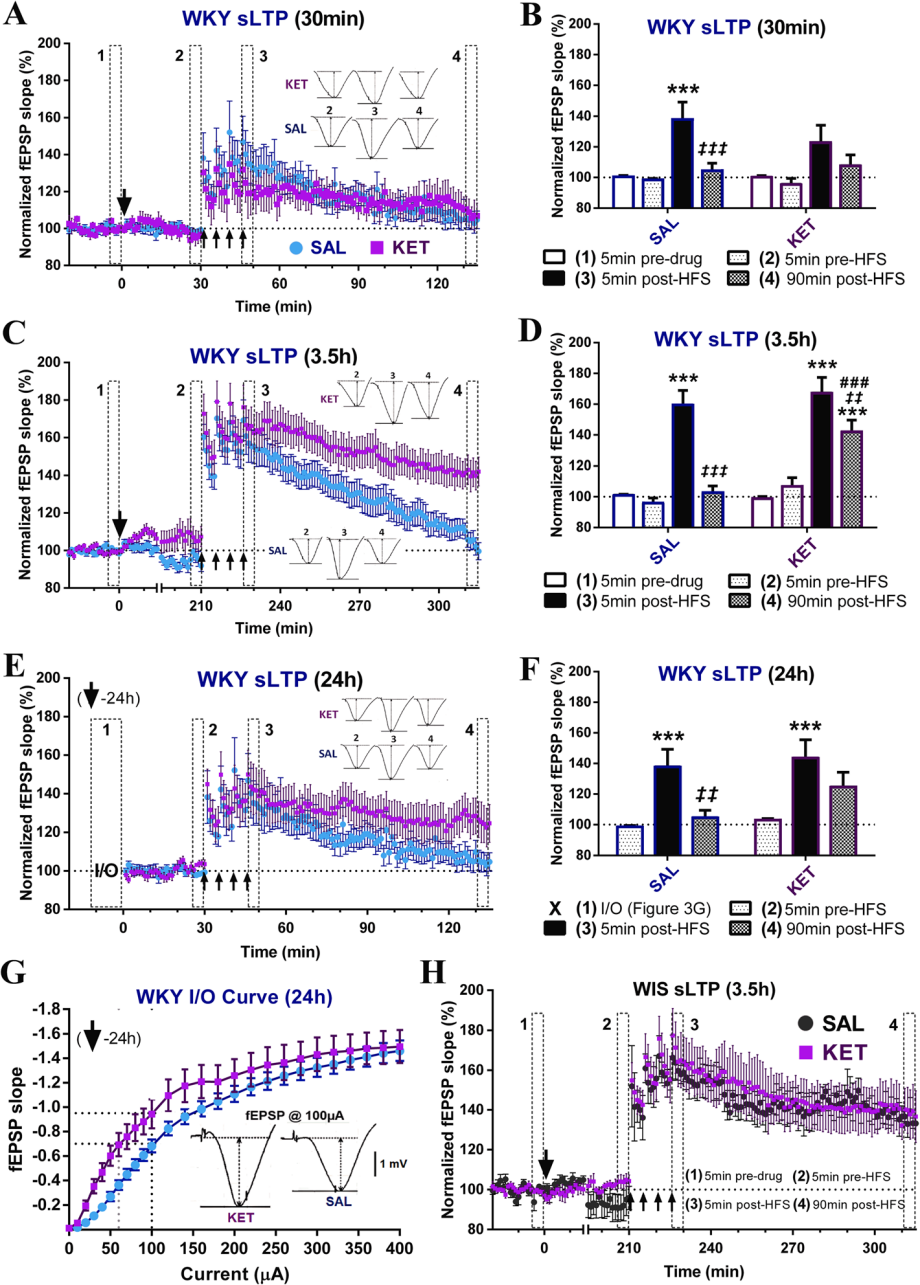
Ketamine restores SC-CA1 sLTP in WKY rats at 3.5 h but not 30 min after injection, with residual effects at 24 h. **a**, **b** WKY saturated LTP at 30 min post-injection by (**a**) drug treatment and (**b**) time bin (SAL: *n* = 12, KET: *n* = 5). Ketamine administration had no significant acute effects on WKY basal synaptic transmission. Although sLTP induction only reached significance in saline (***p < 0.0001) but not ketamine (*p =* 0.14, n.s.) treated rats, there were no significant differences between the two groups at any time point, and no significant sLTP was observed at 90 min as before, regardless of the treatment group (statistically significant decay in SAL rats only, ‡ ‡ ‡*p* = 0.0008). **c**, **d** WKY sLTP at 3.5 h post-injection by (**c**) drug treatment and (**d**) time bin (SAL: *n* = 19, KET: *n* = 18). Initially, significant sLTP was observed in both groups (***p < 0.0001); however, while potentiation again completely decayed in saline-treated WKYs (*‡ ‡ ‡p* < 0.0001), robust sLTP was still present at 90 min post-HFS in the ketamine group following only partial decay (***p < 0.0001; *‡ ‡p* = 0.003; vs. SAL # # #*p =* 0.0003). **e**, **f** WKY sLTP at 24 h post-injection by (**e**) drug treatment and (**f**) time bin (SAL: *n* = 12, KET: *n* = 10). Significant sLTP was induced in both saline (***p = 0.0004) and ketamine (***p = 0.0009) treated WKYs; however, while the potentiation again completely decayed over the 90 min in the saline group (*‡ ‡p* = 0.0024), only partial decay was observed following ketamine (*p =* 0.17, n.s.), so that some sLTP was still present 90 min post-HFS (*p =* 0.097, n.s.). (G) WKY I/O curves at 24 h (SAL: *n* = 12, KET: *n* = 10, same rats as in (**e**, **f**)). fEPSP slope across all current intensities was consistently higher 24 h following ketamine compared to saline treatment, with the stimulation magnitude evoking ~ 50% of the maximal response effectively shifted leftward (from 100 μA to 60 μA) in ketamine-treated rats. **h** WIS sLTP at 3.5 h post-injection by drug treatment (SAL: *n* = 6, KET: *n* = 3). Ketamine had no significant acute effects on WIS basal synaptic transmission. Significant sLTP was observed in both saline *p =* 0.0003) and ketamine *p <* 0.0001) treated WIS rats, and independent of the treatment group, robust sLTP was still present 90 min later (SAL: *p =* 0.028 and KET *p =* 0.013). *Single big arrow = drug injection, 4 small arrows = strong HSF protocol;* * vs. [1] = potentiation effect, *‡* vs. [2] = decay effect, # vs. SAL = treatment effect; *p ≤ 0.05, **p ≤ 0.01, ***p ≤ 0.001

Next, we tested the effects of ketamine (5 mg/kg, ip) on the impaired WKY sLTP at three different time points (30 min, 3.5 h and 24 h) after injection. First, ketamine did not have any significant acute effect on SC-CA1 basal synaptic transmission in WKYs (significant ANOVA main effect of time only, *p* = 0.0002; 5 min pre-drug vs 5 min pre-HFS, Tukey’s *p* = 0.98, n.s.) (Fig. 3a). When the HFS protocol was given 30 min post-injection (Fig. 3a, b), sLTP induction and decay was more pronounced in saline (5 min pre- vs. 5 min post-HFS, Tukey’s *p <* 0.0001) than in ketamine (*p =* 0.14, n.s.) treated rats; however, differences in potentiation between saline and ketamine groups were not significant at any time point (Sidak’s *p >* 0.51, n.s.). In addition, no significant WKY sLTP was observed at 90 min as before, regardless of the treatment group (5 min pre- vs. 90 min post-HFS, Tukey’s *p* > 0.76, n.s.; with statistically significant decay in SAL rats only, 5 min post- vs. 90 min post- HFS, Tukey’s *p* = 0.0008).

When the HFS protocol was given 3.5 h post-injection (significant time x drug treatment ANOVA, *p* < 0.0088) (Fig. 3c, d), sLTP induction itself was not significantly affected by ketamine (5 min pre- vs. 5 min post-HFS, Tukey’s *p <* 0.0001 for SAL and KET; 5 min post-HFS SAL vs. KET, Sidak’s *p =* 0.99, n.s.). Interestingly, regardless of the treatment group, the sLTP induced in WKY rats was more robust when the HFS protocol was given 3.5 h compared to 30 min after injection (Figs. 3c, d and 2e, f), indicating that simply allowing the synapses to recover over a few hours after electrode placement and/or anesthetic administration may facilitate the induction of LTP in this strain. Importantly, however, while sLTP in saline-treated WKY rats again completely decayed (90 min post- vs. 5 min pre- HFS, Tukey’s *p* = 0.75, n.s.; vs. 5 min post- HFS, Tukey’s *p* < 0.0001), significant sLTP was still present in the ketamine treated group at 90 min post-HFS following only partial decay (90 min post- vs. 5 min pre- HFS, Tukey’s *p* < 0.0001; vs. 5 min post-HFS, Tukey’s *p* = 0.003; 90 min post-HFS SAL vs. KET, Sidak’s *p* = 0.0003). Thus, ketamine effectively restored normal SC-CA1 sLTP in WKY rats (Fig. 3c, d), with the magnitude of synaptic potentiation comparable to that observed in normal WIS rats (~ 140% at 90 min; Fig. 2e, f). Consistent with this, at the end of the recording, 13/18 (72%) of ketamine-treated WKY rats (vs. 71% of control WIS rats) and 4/19 (21%) of saline treated WKY rats still expressed robust sLTP at 90 min post-HFS. Therefore, ketamine completely eliminated the SC-CA1 sLTP deficit in WKY rats at 3.5 h after injection, reflecting a robust facilitatory effect on the maintenance of LTP in this model.

When the HFS protocol was given 24 h post-injection (significant ANOVA main effect of time only, *p* < 0.0001), significant sLTP was induced in both saline (5 min pre- vs. 5 min post-HFS, Tukey’s *p* = 0.0004) and ketamine (*p* = 0.0009) treated WKYs, and differences in potentiation between the two treatments did not reach statistical significance at any time point (Sidak’s *p* > 0.22, n.s.) (Fig. 3e, f). However, interestingly, while complete sLTP decay was again observed within 90 min following saline (5 min pre- vs. 90 min post-HFS, Tukey’s *p* = 0.80, n.s., 5 min post- vs. 90 min post-HFS, *p* = 0.0024), only partial decay was observed in the ketamine group (5 min post- vs. 90 min post-HFS, Tukey’s *p =* 0.17, n.s.), so that some sLTP was still present 90 min post-HFS (5 min pre- vs. 90 min post-HFS, Tukey’s *p =* 0.097, n.s.), suggesting some residual drug effect on SC-CA1 sLTP at 24 h after administration. Consistent with this, at 90 min post-HFS, 5/10 (50%) ketamine and only 2/12 (17%) corresponding saline-treated rats still expressed robust sLTP. Although ketamine’s facilitatory effect on sLTP maintenance at 24 h was not as pronounced as at 3.5 h, this treatment did cause a dramatic leftward shift of the WKY SC-CA1 fEPSP slope input-output curve at 24 h (significant ANOVA current intensity main effect and current intensity x drug treatment interaction, *p* < 0.0001 and *p* = 0.004; drug treatment main effect, *p* = 0.14, n.s.) (Fig. 3g). fEPSP slope was consistently higher in the ketamine group at any current intensity, where the stimulation magnitude evoking ~ 50% of the maximal response was effectively shifted leftward (from 100 μA to 60 μA) in rats pre-treated with ketamine compared to saline. The latter effect is consistent with a prolonged enhancement of basal synaptic transmission as a result of sustained ketamine-induced potentiation at this synapse.

Interestingly, under our experimental design, ketamine’s robust synaptic effect was restricted to the WKY SC-CA1 sLTP deficit, as ketamine had no significant effects on sLTP at 3.5 h post-injection in control WIS rats (significant ANOVA main effect of time only, *p* < 0.0001). As in WKYs, ketamine administration had no significant acute effect on WIS basal synaptic transmission (5 min pre-drug vs. 5 min pre-HFS, Tukey’s *p* = 0.96, n.s.); however, there were no significant differences in sLTP between saline and ketamine treated WIS rats at any time point during the recording (Sidak’s *p* > 0.88, n.s.) (Fig. 3h). Significant sLTP was induced in all WIS rats (5 min pre- vs. 5 min post-HFS, SAL Tukey’s *p* = 0.0003 and KET *p* < 0.0001), and independent of the treatment group, robust sLTP was still present 90 min later (5 min pre- vs. 90 min post-HFS, SAL Tukey’s *p* = 0.028 and KET *p* = 0.013). This lack of effect in WIS rats may reflect a sub-optimal ketamine dose (5 mg/kg vs. usual 10 mg/kg), the single time point tested (3.5 h post-ketamine) and/or a LTP ceiling effect, as the strong HFS protocol may have elicited close to maximum levels of LTP (i.e. saturated LTP, precluding any further drug-induced synaptic potentiation in the control strain).

### Ketamine restores long-term spatial memory in WKY rats

**Fig. 4:**
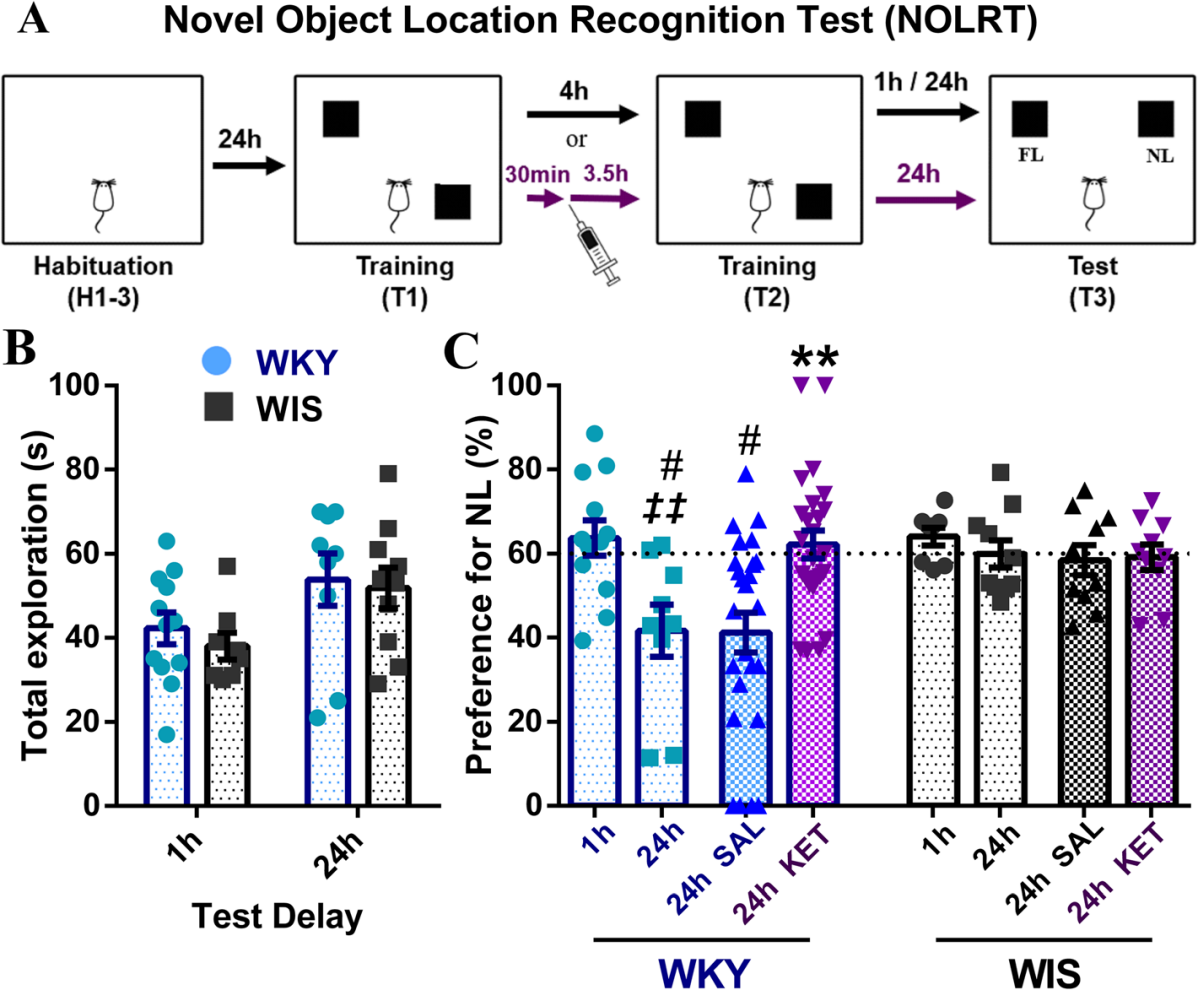
Ketamine restores NOLRT long-term spatial memory in WKY rats, without affecting performance in WIS controls. **a** In the NOLRT, following 3 days of habituation (H1–3), rats received 2 training sessions (T1 and T2, 10 min each), with two identical objects placed in two opposing arena corners. During the testing session (T3, 1 h or 24 h later for short or long -term memory), one object stayed at the familiar location (FL), while the other was moved to a new location (NL), where an NL preference (NL/NL + FL) of 60% or more indicates strong hippocampal-dependent spatial memory. In experiments involving drug treatment (purple arrows), saline, ketamine or (2R,6R)-HNK were injected 3.5 h before T2, i.e. 27.5 h before the 24 h testing session (T3). **b** Total NOLRT T3 exploration time (NL + FL) at 1 h or 24 h (*n* = 8–12/group) was comparable between the two strains. **c** % NL preference for the NOLRT test session for drug-free (1 h or 24 h) and drug-treated (saline or ketamine) WKY and WIS rats. Under drug-free conditions (1 h/24 h drug-naïve: *n* = 8–12/strain, same rats as in (**b**)), short-term memory (at 1 h) was equivalent between strains; however, long-term spatial memory (at 24 h) was significantly impaired in WKY compared to WIS rats (#*p* = 0.027; WKY 1 h vs. 24 h *‡ ‡p* = 0.0037). In drug-treated rats (24 h SAL/KET WKY: *n* = 25/group and WIS: *n* = 10/group), while NOLRT location recognition memory at 24 h remained impaired in saline treated WKY compared to WIS rats (#*p* = 0.05), ketamine administration significantly facilitated long-term spatial memory compared to saline-treated WKYs (***p = 0.0008), effectively restoring performance to control WIS levels (~ 60% at 24 h). Ketamine had no effect on NOLRT performance in WIS controls. # vs. WIS = strain effect, *‡* drug-naïve 24 h vs. 1 h = time delay effect, * vs. SAL = treatment effect; *p ≤ 0.05, **p ≤ 0.01, ***p ≤ 0.001

To further investigate the functional effects of ketamine in this model, we sought evidence for a functional correlation between SC-CA1 LTP and cognitive performance on a test of spatial memory, mediated by activity in the dHPC [31–33]. To this end, we compared performance of WKY and WIS rats on the novel object location recognition task (NOLRT, Fig. 4a), where preference of 60% or more for the object at the new location (NL) during the testing session (either 1 h or 24 h after training for short or long -term memory, respectively) indicates strong hippocampal-dependent spatial memory. Total exploration time during the test session (1 h or 24 h) was comparable between the two strains (significant ANOVA main effect of strain only, *p* = 0.010, but no post-hoc pairwise significance, Sidak’s *p* > 0.77, n.s.) (Fig. 4b). Importantly, in the NOLRT (significant strain by test delay ANOVA, *p* < 0.047) while short-term location recognition memory (at 1 h, Fig. 4c) in the task was equivalent between the strains (Tukey’s *p* > 0.99, n.s.), long-term memory (at 24 h, Fig. 4c) was significantly impaired in WKY compared to WIS rats (Tukey’s *p* = 0.027; WKY 1 h vs. 24 h, Tukey’s *p* = 0.0037), consistent with the SC-CA1 LTP deficit in these animals. Next, while 24 h NOLRT location recognition memory remained impaired in saline treated WKYs (vs. 24 h SAL WIS, Tukey’s *p* = 0.05), ketamine significantly facilitated long-term object recognition memory in the WKY rat compared to saline-treated WKYs (significant ANOVA drug treatment main effect and strain x drug interaction, *p* = 0.03 and *p* = 0.04; WKY 24 h SAL vs. KET, Tukey’s *p* = 0.0008). Importantly, ketamine administration effectively restored WKY NOLRT performance to control WIS levels (~ 60%), consistent with ketamine’s positive effects on WKY dHPC synaptic plasticity. In contrast, ketamine had no significant effects on NOLRT performance in control WIS rats (trend towards ANOVA main effect of strain, *p* = 0.15, n.s., 24 h SAL vs. KET, Tukey’s *p* = 0.99, n.s.) (Fig. 4c).

### (2R,6R)-HNK restores normal SC-CA1 sLTP and long-term spatial memory without affecting FST immobility in WKY rats

**Fig. 5:**
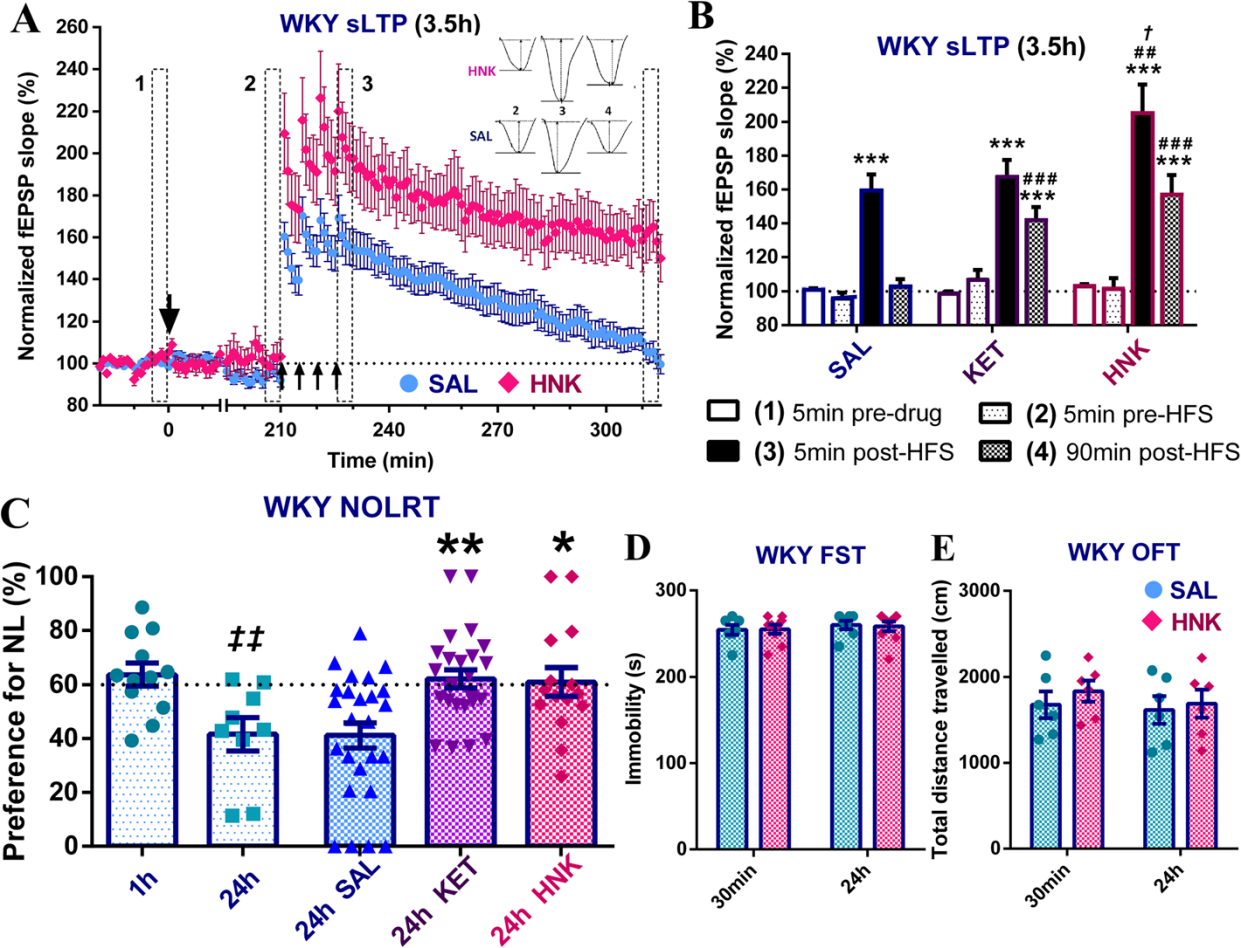
(2R,6R)-HNK restores SC-CA1 sLTP and NOLRT long-term spatial memory without affecting FST immobility in WKY rats. **a**, **b** WKY sLTP at 3.5 h post-injection by (**a**) drug treatment and (**b**) time bin (SAL: *n* = 19, HNK: *n* = 8, +KET: *n* = 18 in (**b**) only). (2R,6R)-HNK had no significant acute effects on WKY basal synaptic transmission. Significant sLTP was initially present in all groups (***p < 0.0001); however, potentiation immediately post-HFS was significantly higher in HNK treated rats compared to saline # #*p* = 0.0015) but also ketamine (*†p* = 0.017). In addition, while significant decay was seen in all groups (*p* ≤ 0.003, *not shown*), robust sLTP was still present 90 min later in the HNK-treated group (***p < 0.0001, vs. SAL # # #*p* < 0.0001), which is now similar in magnitude to that following ketamine, with no sLTP in the saline group as expected. *Single big arrow = drug injection, 4 small arrows = strong HSF protocol;* * vs. [1] = potentiation effect, # vs. SAL and *†* vs. *KET* = treatment effects; **c** WKY % NL preference for the NOLRT test session (1 h/24 h drug-naïve: *n* = 9–12/group; SAL: *n* = 25, KET: *n* = 25 and HNK: *n* = 15). The WKY NOLRT long-term memory deficit (drug-naïve 24 h vs. 1 h, *‡ ‡p* = 0.0037) was significantly reversed by (2R,6R)-HNK (vs. SAL *p = 0.012), as previously reported with ketamine (**p = 0.0017). *‡* drug-naïve 24 h vs. 1 h = time delay effect, * vs. SAL = treatment effect; **d** Average day2 FST immobility for saline (*n* = 7/group) and (2R,6R)-HNK (*n* = 9/group) treated WKY rats at 30 min and 24 h post-injection, indicating no effects of HNK in the FST. **e** Average total distance travelled in the OFT for saline and (2R,6R)-HNK treated WKY rats (*n* = 6/group) at 30 min or 24 h post-injection, where HNK had no effects on WKY general locomotor activity. *p ≤ 0.05, **p ≤ 0.01, ***p ≤ 0.001

Similar to ketamine, the (2R,6R)-HNK metabolite (5 mg/kg, ip) did not have significant acute effects on SC-CA1 basal synaptic transmission in WKY rats (5 min pre-drug vs 5 min pre-HFS, Tukey’s *p* = 0.99, n.s.) (Fig. 5a). Furthermore, like its parent drug, (2R,6R)-HNK also had a pronounced facilitatory effect on WKY SC-CA1 sLTP at 3.5 h post-injection (significant drug treatment by time ANOVA, *p* < 0.0088) (Fig. 5a, b). Significant sLTP was induced in all groups (5 min pre- vs. 5 min post-HFS, Tukey’s *p* < 0.0001); however, potentiation immediately post-HFS was significantly enhanced in HNK compared to saline (Sidak’s *p* = 0.0015) but also ketamine (*p* = 0.017) treated WKYs. In addition, despite significant decay in all groups (5 min post- vs. 90 min post- HFS, Tukey’s *p* ≤ 0.003), potentiation was again lost in the saline group (5 min pre- vs. 90 min post-HFS, Tukey’s *p* = 0.75, n.s.), while robust sLTP was still present 90 min later in HNK-treated WKYs (5 min pre- vs. 90 min post-HFS, Tukey’s *p* < 0.0001; 90 min post-HFS SAL vs. HNK, Sidak’s *p* < 0.0001), which was now similar in magnitude to that following ketamine (90 min post-HFS KET vs. HNK, Sidak’s *p* = 0.93, n.s.). At the end of the recording, 7/8 (88%) of HNK-treated WKY rats still expressed robust sLTP, compared to 72% for ketamine and 21% for saline. Consistent with these effects on synaptic plasticity, (2R, 6R)-HNK pre-treatment also significantly restored WKY long-term (24 h) object location recognition memory in the NOLRT (significant ANOVA effect of drug treatment, *p* = 0.001) (Fig. 5c). The WKY NOLRT long-term memory deficit (1 h vs. 24 h, Tukey’s *p* = 0.0037) was significantly reversed by (2R, 6R)-HNK compared to saline (Tukey’s *p* = 0.012), as previously seen with ketamine (*p* = 0.0017). Interestingly, in the WKY strain, we failed to detect any activity of (2R,6R)-HNK at either 30 min or 24 h after injection using the FST (Fig. 5d) (no significant ANOVA main or interaction effects, *p* > 0.42, n.s.) or OFT (Fig. 5e) (no significant ANOVA main or interaction effects, *p* > 0.45, n.s.).

## Discussion

Recent studies have reported several abnormalities in synaptic plasticity processes within key neural circuits relevant to depression in the WKY model [34–40], which not only provide support for our findings, but further highlight the utility of this strain for the study of synaptic plasticity changes and their contribution to key depressive-like phenotypes and antidepressant responses (for review, [7]). Overall, reductions in total hippocampal volume and impairments in LTP at both medial perforant path – dentate gyrus (mPP-DG) and SC-CA1 synapses in the stress-prone WKY rat appear to reflect a global impairment of hippocampal synaptic plasticity and function [35, 36, 38, 40]; however, to our knowledge, the present experiments constitute the first systematic study of hippocampal synaptic plasticity (LTD, weak and strong LTP) in this model. Consistent with findings from stress-based models of depression, the normal balance in hippocampal synaptic plasticity is innately perturbed in the WKY rat [7, 35, 40]. Since LTP, which facilitates spine formation/enlargement, is significantly impaired, whereas LTD, associated with spine shrinkage/retraction, appeared to be unaffected in the WKY HPC, there may be a higher propensity toward synaptic destabilization, loss of connectivity and eventually, neuronal atrophy in this key neuronal circuit implicated in MDD, possibly mediating or at least contributing to the structural and functional findings in the WKY strain [3, 7, 11, 13, 15, 41].

On the other hand, accumulating evidence suggests that ketamine may reverse the stress-induced impairment of connectivity by engaging synaptic plasticity and synaptogenesis to “reset the system” (for review, [7]). It should be noted, however, that ketamine’s effects on in vivo synaptic plasticity in the HPC and beyond, and their direct contribution to its antidepressant action, are still unclear. To address this, we first successfully replicated ketamine’s significant rapid (30 min) and sustained (24 h) antidepressant-like effects in both WKY and WIS rats using the FST, the most commonly used preclinical screen for antidepressant activity [21, 36, 42–48]. Based on ketamine’s mechanism of action (direct NMDAR inhibition and indirect AMPAR facilitation) and the unique roles of NMDA and AMPA receptors in the induction and expression of synaptic plasticity, respectively, it is crucial to consider the temporal effects of ketamine treatment on hippocampal LTP (i.e. both its acute and delayed drug effects). Importantly, we found that ketamine acutely restored the impaired WKY SC-CA1 LTP at 3.5 h but not 30 min after injection, with a residual LTP facilitatory effect and an overall increase in basal synaptic transmission at 24 h. Although the literature is limited in the context of the WKY model, effective antidepressant treatments, including ketamine, electroacupuncture or even certain protocols of electrical stimulation, have now been reported to restore dHPC LTP in this strain, thereby restoring hippocampal memory function and reversing the associated behavioural deficits in WKY rats [7, 35, 36, 38, 40]. Interestingly, Belujon and Grace previously demonstrated that ketamine restores escape behaviour, as well as LTP in the hippocampus-accumbens pathway, in WKY rats subjected to a learned helplessness paradigm [34].

While antidepressant doses of ketamine do not generally affect hippocampal basal transmission [49–51] as observed here, accumulating evidence suggests that ketamine may selectively modulate synaptic plasticity processes. There have been numerous, often conflicting, reports on the effects of ketamine on synaptic plasticity in normal outbred rats, with many studies showing acute inhibition of hippocampal LTP [51–53] and/or LTD [49, 51, 54], effects which seem to be in line with ketamine’s action as an open-channel NMDAR antagonist. However, importantly, others have previously reported no change [49, 50] or even a facilitation of LTP [55–57] in the rodent HPC following ketamine. Collectively, these studies suffer from important inconsistencies and limitations, such as utilizing mostly in vitro electrophysiological recordings using hippocampal brain slices. Other factors that may contribute to the discrepancies seen in the literature include different doses and routes of ketamine administration (e.g. systemic injections of 10-100 mg/kg or bath application of 10-100 μM onto slices), different (often single) time points after ketamine administration, different LTP/LTD protocols and strains of outbred rats. Previous studies reporting LTP inhibition following ketamine (e.g. 51–53) examined synaptic plasticity under conditions where NMDARs are indeed blocked, namely post-administration with drug on board and/or after relatively high doses of ketamine (10-100 mg/kg or 10-100 μM versus 5 mg/kg in the present study). Importantly, these doses often greatly exceed peak antidepressant ketamine doses/concentrations reported in the context of depression (< 1 μM in humans following 0.5 mg/kg, IV or ~ 5 μM in mice after 10 mg/kg, ip [21, 58]).

Under our experimental design, ketamine may have failed to affect sLTP in control WIS rats, due to a sub-optimal ketamine dose (5 mg/kg vs. > 10 mg/kg in the literature), the single time point tested (3.5 h post-ketamine) and/or a ceiling effect (i.e. nearly saturated LTP induced by the strong HFS protocol). Although additional studies are needed to better characterize the effect of ketamine (e.g. 5-10 mg/kg at 30 min-days post-injection) on LTP in outbred rats, accumulating data indicate that ketamine can facilitate non-saturated LTP at lower doses and/or longer post-injection delays in rat strains other than inbred WKY rats. Specifically, two previous studies confirm that ketamine (3–10 mg/kg, IV) enhances SC-CA1 fEPSP wLTP following sub-maximal HFS in hippocampal slices obtained from normal outbred rats at 24 h post-injection [55, 56], similar to our observations in stress-prone WKY rats. Moreover, one study found that chronic social defeat stress applied to mice impairs SC-CA1 LTP in vitro, which was reversed by ketamine (5 mg/kg, ip) at 24 h post-injection [57]. Clearly, as indicated by the present study, there is a need for systematic investigations of ketamine’s effects on in vivo hippocampal synaptic plasticity at therapeutically relevant doses, at appropriate post-injection intervals and in the context of valid preclinical models of depression.

Initially, ketamine’s action as an antidepressant was simply attributed to an acute blockade of the NMDA receptor, giving rise to the NMDAR inhibition hypothesis of ketamine’s effects on depression. This hypothesis, in turn, prompted the evaluation of alternative NMDAR antagonists as novel, safer antidepressants. Disappointingly, human clinical trials failed to confirm that alternative NMDAR blockers (e.g. memantine, AZD6765 and CP-101,606) shared ketamine’s robust, rapid and/or sustained antidepressant effects. Indeed, to date, ketamine is the only NMDAR antagonist to consistently demonstrate antidepressant efficacy in multiple trials [21, 59, 60]. Preclinical studies also report that treatment with the NMDAR antagonists MK-801 and memantine, which bind to the same receptor site as ketamine, lack sustained antidepressant effects in rodents [21, 61]. Subunit composition of heteromeric NMDARs, namely the presence of GluN2A versus GluN2B subunits, is another important consideration. Specifically, low doses of ketamine have been proposed to selectively block GluN2B-containing NMDARs that may be 1) tonically activated by spontaneously released and/or ambient glutamate, and 2) mainly extra-synaptic and potentially more accessible to exogenous antagonism [62–65]. Higher doses of ketamine may gradually inhibit synaptic NMDARs containing more GluN2A subunits involved in the induction of LTP, thereby mediating the acute inhibitory effect of ketamine on LTP [62]. This body of work has prompted the hypothesis that while ketamine is non-subunit specific, antagonism of GluN2B-containing NMDARs might be responsible for its antidepressant action [62, 63, 66]. In partial support of this conjecture, the selective GluN2B antagonist, Ro25–698 possesses rapid antidepressant action in rodents; however, the effects appear to be less robust and/or shorter-lasting compared to ketamine [21, 61, 67–69]. Finally, while initial clinical studies usually employed the racemic form of ketamine, the enantiomer R-ketamine, which is ~ 4 times less potent at inhibiting NMDAR than S-ketamine, has a more potent and sustained antidepressant action in rodents [60, 70]. Overall, the differential clinical action of NMDAR blockers can be attributed to a broad range of factors related to NMDAR antagonism, such as specificity in terms of receptor subunit and localization, extent/nature of channel block including drug affinity, trapping, receptor state dependence, competitive/non-competitive properties, etc., as well as the unique downstream effects of the acute drug-receptor interaction [19]. Unlike other members of this drug class, ketamine is a non-competitive, open-channel antagonist, which binds to the NMDAR with a low affinity and high but not complete trapping (86%), and is uniquely associated with the induction of long-lasting, NMDAR inhibition-dependent synaptic plasticity thought to underlie its robust and sustained antidepressant effects [19, 21, 63].

It is becoming increasingly clear that it is crucial to consider not only ketamine’s unique acute action on the NMDA receptor, but also the key downstream signaling events as a result of ketamine-induced NMDAR inhibition (e.g. suppression of eukaryotic elongation factor 2 (eEF2)-mediated protein synthesis and recruitment of BDNF- and mTOR- dependent synaptogenic pathways), effects which develop over time and are not observed with alternative NMDAR antagonists lacking antidepressant properties [19, 21, 63]. While the specific mechanisms underlying ketamine’s facilitatory effects on LTP observed here remain unknown, many factors must be considered given ketamine’s unique abilities to modulate specific NMDAR and AMPA receptor subtypes, release of glutamate and monoamines, along with effects on expression of key post-synaptic proteins involved in the regulation of synaptic plasticity (for review, see [7]). One possible mechanism that deserves particular consideration is the atypical protein kinase C isoform, protein kinase Mζ (PKMζ), which plays an essential role in LTP maintenance and memory retention in various brain regions, including the HPC [31–33]. Previous studies have revealed the regulatory effects of PKMζ on the expression and localization of synaptic proteins, especially AMPARs, which ultimately counteract the active decay of LTP and maintain synaptic potentiation and memory retention [31–33]. Interestingly, a recent study reported that chronic mild stress increased depressive-like and anxiety-like behaviors, decreased the expression of PKMζ in the rodent mPFC and HPC, and induced synaptic deficits that were reversed or mimicked by PKMζ overexpression or inhibition, respectively [71]. Importantly, PKMζ synthesis is regulated by multiple kinases implicated in ketamine’s actions, including mTOR, and to complete this intriguing sequence, mPFC PKMζ expression is increased by antidepressant doses of ketamine [71]. Accordingly, PKMζ warrants further investigation as a potential critical mediator of depressive-like behavior, synaptic plasticity across different brain regions and antidepressant effects of novel drugs including ketamine [71].

As mentioned, previous work highlights the critical role of dHPC LTP in long-term spatial memory persistence and its decay, induced by experimental reduction of synaptic expression of AMPARs [31–33]. This forgetting process, which actively erases consolidated long-term memories in the HPC and other brain structures, is normally tightly regulated and contributes to establishing adaptive behavior. Importantly, this cognitive function may be dysregulated in the context of neuropsychiatric disorders such as depression, promoting the associated decline of memory and cognition [14, 31–33]. Accordingly, we examined whether the impaired SC-CA1 LTP observed in WKY rats may be associated with accelerated the forgetting of long-term spatial memory, and if so, whether this cognitive deficit may be rescued by ketamine. Consistent with the SC-CA1 LTP deficit and corresponding positive effects of ketamine, WKY rats exhibited a selective deficit in hippocampal-dependent long-term spatial memory (as measured by the NOLRT at a delay of 24 h), and importantly, this striking deficit was effectively eliminated by ketamine pre-treatment. Accordingly, our results indicate that by restoring sLTP in stress-prone WKYs, ketamine treatment rescued a long-term object location memory deficit reminiscent of certain cognitive impairments that are a key feature of depression.

It is well established that stress, including learned helplessness, chronic mild stress, chronic social defeat or even aging, can impair hippocampal LTP in outbred rats, which in turn is associated with various cognitive impairments, including spatial memory deficits, in addition to other depressive-like phenotypes [72–78]. Although assessment of cognitive/memory function in the WKY rat model of depression is sparse, available reports indicate significant impairments in spatial and object recognition memory in this strain [40, 79–81]. We have argued that the stress-induced dysregulation of hippocampal synaptic plasticity and associated memory processes may be an innate characteristic of the WKY model [7], which can be effectively eliminated by ketamine as observed here. Interestingly, in WKY rats, electroacupuncture exerts antidepressant-like activity in the FST, while also restoring in vitro SC-CA1 LTP and HPC-dependent spatial memory [40]. Similarly, various pharmacological and environmental interventions (e.g. GLYX-13/rapastinel and environmental enrichment) reverse stress-induced deficits in hippocampal LTP and associated spatial memory in outbred rats [77, 78, 82–85]. Together these many and varied findings strengthen the link between hippocampal synaptic plasticity and memory dysfunction in the context of stress/depression.

In common with its parent drug, we show (2R,6R)-HNK (5 mg/kg, ip) also effectively restored normal SC-CA1 sLTP in WKY rats at 3.5 h post-injection. HNK induces rapid and sustained enhancement of AMPAR function via a mechanism that does not involve NMDAR inhibition (for review, [19]). Importantly. current findings with the WKY model indicate that the effects of both ketamine and its metabolite are mediated by selective modulation of metaplasticity processes (i.e., the activity-dependent modulation of subsequently induced synaptic plasticity, in this case, LTP), without affecting basal synaptic transmission. Consistent with its positive effects on SC-CA1 LTP, in the present study, (2R,6R)-HNK also rescued hippocampal-dependent long-term object location memory in WKYs. Interestingly, (2R,6R)-HNK failed to reverse the FST deficit in WKY rats. As discussed, there are conflicting preclinical reports about the antidepressant efficacy of HNK (at 10 mg/kg, ip) [21–25]. It is possible that the metabolite did not affect FST immobility in our study because the WKY rat represents a relatively severe, treatment-resistant model of depression [7], although positive effects at higher doses (> 5 mg/kg) and/or in more valid preclinical tests of depression cannot be ruled out in the context of the WKY model. Based on our findings and those of others [19, 21–25], it appears that at equivalent doses, (2R,6R)-HNK recapitulates some, but not all, aspects of ketamine’s molecular and antidepressant effects. Accordingly, consideration should be given to more precise dissection of mechanistic points of overlap and divergence in the actions of ketamine and this key active metabolite.

The observation that (2R,6R)-HNK failed to decrease FST immobility in WKY rats despite restoring SC-CA1 LTP, raises important questions about the role of LTP at this synapse in accounting for the effects of antidepressants in the FST. Based on these findings, we propose that in the WKY model of depression, pharmacological restoration of dHPC synaptic plasticity does not underlie ketamine’s effects in the FST. Instead, we propose that the common ability of both ketamine and (2R,6R)-HNK to restore SC-CA1 LTP mediates reversal of hippocampal-dependent cognitive deficits, such as spatial memory, which are also key features of clinical depression. Overall, our results highlight the potential pro-cognitive action of ketamine and HNK in the context of depression, as well as the role of hippocampal synaptic plasticity in the development and reversal of certain, more cognitive, depressive-like phenotypes.

Clinical studies report that hippocampal volume loss in depression predicts illness duration and severity, and is associated with well-documented cognitive deficits and poorer clinical outcomes [1, 4, 14, 15, 36, 86–90]. Therefore, a pronounced hippocampal deficit, as in the case of both WKY rats and patients suffering from mood and anxiety disorders, can result in impairments in various aspects of hippocampal-dependent learning, rendering an individual more vulnerable to developing symptoms of depression and/or anxiety, as well as contributing to the complex cognitive deficits observed in depression [1, 4, 14, 15, 36, 86–89, 91]. Given that substantial aspects of the burden of depression are attributed to these cognitive impairments, their effective treatment by novel antidepressants such as ketamine and related compounds holds promise of improved long-term outcomes and functional recovery [92].

Concern has been raised that ketamine, at high concentrations and/or repeated schedules of administration, or when the drug is on board during task performance, may impair hippocampal function and/or have non-specific side effects (e.g. attentional, sensory-motor, etc.), subsequently disrupting spatial learning and memory [93–101]. Indeed, mild and transient psychotomimetic side effects and cognitive deficits are commonly reported in the clinic during and shortly after ketamine infusion. However, when doses and time points are selected to ensure ketamine has positive effects on hippocampal synaptic plasticity and function, especially in the context of models of hippocampal dysfunction (e.g. WKYs, stressed/aged outbred rats), then ketamine treatment may have pro-cognitive effects, as observed here. Consistent with this, positive effects of low-dose ketamine on cognitive function in rodents are numerous [12, 44, 102, 103], with one study, for example, reporting that ketamine (5 mg/kg, ip) can restore SC-CA1 LTP and associated spatial working memory at 24 h in mice subjected to chronic social defeat [57]. Importantly, recent clinical trials indicate that ketamine’s sustained antidepressant effects in TRD patients appear to be accompanied by significant improvements in cognitive function, highlighting a promising future research avenue [104–106].

Although ketamine’s ability to restore hippocampal-dependent function by modulating synaptic plasticity is a plausible mechanism for some of its therapeutic effects related to functions mediated by the dHPC, it is highly unlikely that such localized effects can account for ketamine’s wide range of antidepressant effects. Indeed, it is becoming increasingly clear that ketamine has unique effects on multiple neural circuits implicated in depression that likely mediate different aspects of its antidepressant activity [7].. In summary, dHPC synaptic plasticity may represent a neural substrate for ketamine’s effects on more cognitive, hippocampal-dependent functions, whereas other circuits (e.g. ventral HPC, PFC, amygdala, mesolimbic system) likely mediate positive treatment effects on other facets relevant to depression, including stress coping, anhedonia and emotional dysregulation.

Based on experience gained from the present study, we recommend several strategies to better understand the neural bases and unique clinical properties of novel, rapid-acting antidepressants. The first approach involves greater reliance on animal models of heightened stress susceptibility and resistance to conventional antidepressant drugs (e.g. the WKY rat), in order to increase the relevance for vulnerable and treatment-resistant populations. Next, we emphasize the importance of deconstructing different depression-like phenotypes and corresponding antidepressant responses by utilizing more comprehensive batteries of preclinical tests across various domains affected in MDD (e.g. stress/emotional reactivity, motivation and cognition). Moving beyond an over-reliance on predictive tests such as the FST, more precise and reliable measures of specific aspects of depression permit better identification of the neural circuits mediating the development and reversal of specific behavioural phenotypes of interest. This continued effort to redefine clinical symptoms of depression in terms of brain circuit dysfunctions will enhance our understanding of the pathophysiology underlying various symptoms of depression, and may provide novel strategies for enhancing antidepressant efficacy and selectively targeting different symptoms/endophenotypes of MDD.

## Supplementary information

**Additional file 1. **Supplementary methods and results, including supplementary figures (**Figure S1A**. ^1^H NMR spectrum of (2R,6R)-HNK.** Figure S1B.**^13^C NMR spectrum of (2R,6R)-HNK).

## Data Availability

Key data supporting the conclusions of this article are included within the article and its supplementary information file. The datasets used and/or analysed during the current study are available from the corresponding author on reasonable request.

## Abbreviations

AMPAR: α-amino-3-hydroxy-5-methyl-4-isoxazolepropionic acid receptor
ANOVA: Analysis of variance
BDNF: Brain-derived neurotrophic factor
DG: Dentate gyrus
dHPC: Dorsal hippocampus
fEPSP: Field excitatory post-synaptic potentials
FST: Forced swim test
HFS: High frequency stimulation
HNK: (2R,6R)-hydroxynorketamine
HPA: Hypothalamic-pituitary-adrenal
HPC: Hippocampus
KET: Ketamine
LFS: Low frequency stimulation
LTD: Long-term depression
LTP: Long-term potentiation
MDD: Major depressive disorder
mPP: Medial perforant path
mTOR: Mammalian target of rapamycin
NMDAR: N-methyl-D-aspartate receptor
NOLRT: Novel object location recognition test
PFC: Prefrontal cortex
PKMζ: Protein kinase Mζ
SAL: Saline
SC: Schaffer collateral
SEM: Standard error of the mean
sLTP: Strong LTP
SSRI: Selective serotonin reuptake inhibitor
TRD: Treatment-resistant depression
WIS: Wistar
WKY: Wistar-Kyoto
wLTP: Weak LTP
