# Supplementary material for: Ketamine and its metabolite, (2R,6R)-HNK, restore hippocampal LTP and long-term spatial memory in the Wistar-Kyoto rat model of depression
Source: Mol Brain. 2020 Jun 16;13:92. doi: 10.1186/s13041-020-00627-z (PMC7296711; doi:10.1186/s13041-020-00627-z)
Supplement: Supplementary file 1 — Additional file 1. Supplementary methods and results, including supplementary figures (Figure S1A. 1H NMR spectrum of (2R,6R)-HNK. Figure S1B.13C NMR spectrum of (2R,6R)-HNK). [file 13041_2020_627_MOESM1_ESM.docx]

**Aleksandrova et al., *Molecular Brain, 2020* (MBRJ-D-20-00072)**

**Ketamine and its Metabolite, (2R,6R)-HNK, Restore Hippocampal LTP and Long-Term Spatial Memory in the Wistar-Kyoto Rat Model of Depression**

### Supplementary Information

### 1. Supplementary Methods

#### Behavioural Testing

***Open field test (OFT)***

The open field test (OFT) is used to assess an animal’s exploration of a novel environment (an open-field arena) by measuring spontaneous locomotor activity (i.e. total distance traveled, velocity, number of rearings, time spent in center of the arena, etc.). Eight black plexiglass boxes measuring 41.25cm * 41.25cm * 41.25cm were used as arenas. Red LED lights placed above the boxes provided lighting and the room was otherwise held in darkness. OFT testing was performed without habituation to the testing apparatus. Locomotor activity within each arena was tracked by two overhead video cameras and scored with Ethovision XT (Noldus), which was set to track 10min of locomotor activity upon detection of movement within each arena zone. Following testing completion, the absence of tracking errors was verified using the stored video recording. In experiments involving drug treatments, saline, ketamine or HNK were injected either 30min or 24h before OFT testing.

***Forced Swim Test (FST)***

The forced swim test (FST) is used to measure abnormal stress coping in rats and as a screen for antidepressant-like drug action. Briefly, rats were individually placed into a clear plastic cylinder (20cm in diameter, 50cm tall) filled with room temperature water (25±0.5 °C) at a height of 30cm to ensure that animals could not touch the bottom of the container with their hind paws or tails. Animals underwent a 15min pre-exposure FST session on day 1, followed by a 5min test session on day 2. FST test sessions were recorded using a video camera and manually scored later. After the FST, rats were dried and placed in a recovery chamber under a heat lamp for 10min before being returned to their home cage, and the water was changed between each animal. A time-sampling scoring technique was used, whereby the predominant behavior (immobility or swimming) in each 5s period of the 300s test was recorded. The definition of immobility in the FST is that the rat remained floating in the water without struggling and made only movements necessary to keep its head above the water. We included the climbing score with the swimming as an active behavior because very few such incidences were observed. Scoring was done by an experienced experimenter blind to the experimental condition. In experiments involving drug treatments, saline, ketamine or HNK were injected either 30min or 24h before FST test on day2.

***Novel Object Location Recognition Test (NOLRT)***

In order to avoid possible confounding factors associated with other spatial memory tasks (e.g. reliance on locomotion, aversion or reward for task performance may not be compatible with the WKY model), we chose to assess hippocampal-dependent spatial memory using the novel object location recognition test (NOLRT), an emotionally neutral task that involves unrestricted exploration in the absence of any external reinforcement or punishment, which instead exploits rodents’ natural curiosity and innate preference for novelty (1, 2). This task is quite similar to procedures used in humans and should therefore have good predictive validity (3). The NOLRT allows for measurement of both short-term (at a delay of 1h between training and testing) and long-term (at a delay of 24h) spatial memory (2). In the task, animals were habituated to an open field arena (60 x 60 x 60cm) placed in a room with external cues, for 3 consecutive days (10min sessions). They were then given 2 training sessions on day 4 (T1 and T2, 10min each, morning and afternoon), where two identical objects (Lego figures mounted to the arena floor) were placed in the arena in a certain spatial configuration (i.e. in two opposite corners of the apparatus, 10cm from the sidewall, with starting locations counterbalanced between different experimental groups to reduce potential biases due to preferences for particular locations). Rats were placed in the middle of the apparatus and were left to explore these two identical objects for 10min. After T1, rats were put back in their home cages and an ITI of ~3h was given before subjecting them to a second training session (T2) as before (with objects in the same locations at in T1). During the testing session (T3, either 1h or 24h later), one of the objects remained at the familiar location (FL), while the other was moved to a new location (NL), and rats were again allowed to explore freely for 10min. The apparatus and the objects were thoroughly cleaned after each trial to avoid the presence of olfactory trails. Sessions T1-3 were recorded using a video camera and the time spent exploring each object location were determined only for the first 2min of each session by manual scoring of the video recordings, done by an experienced experimenter blind to the experimental condition. Object exploration was defined as the time when the rat's head was oriented toward the object within 45 degrees and was within 3cm of the object. An NOLRT preference of around/above 60% for the object that has been moved to the novel location on the test day (NL/NL+FL) indicates good spatial recognition memory (since animals are drawn to explore novelty). In experiments involving drug treatments, saline, ketamine or HNK were injected 3.5h before the second training session on day 4, or 27.5h before testing on day 5 (time point chosen based on our electrophysiology data).

#### *In vivo* Electrophysiology

*In vivo* extracellular recordings were used to measure and compare hippocampal basal synaptic transmission, as well as synaptic plasticity (LTP and LTD), between the two strains (WKY and WIS) and under the different treatment conditions (drug-free, saline, ketamine or HNK administered to different groups of rats). The *in vivo* electrophysiological recordings were conducted using techniques described previously (4). On the day of the experiment, rats were removed from the colony room, weighed and immediately anesthetized (using urethane, 1.5g/kg, ip, for non-recovery experiments). Urethane for *in vivo* electrophysiology experiments was obtained from Sigma-Aldrich (St. Louis, USA), dissolved in distilled water and injected ip at a dose of 1.5g/kg. Where necessary, a supplemental dose of anesthesia (urethane in increments of 0.3ml at a time) was administered as appropriate. Once fully unconscious, they were placed in a stereotaxic frame, and gel tears applied to their eyes. The animals' body temperature was controlled with a regulated heating pad set at 36.8°C. The degree of anesthesia was monitored through respiratory rate and withdrawal reflex. Using sterile techniques, an incision was made along the dorsal surface of the skull to expose the landmark bregma. Two holes (1-2mm in diameter, for the recording and stimulating electrodes, respectively) were drilled with a stereotaxic drill above the brain structures of interest (in this case the hippocampus). One hole was drilled posterior and lateral to bregma for the recording electrode ground wire, and one over the left frontal lobe for a single reference electrode. A bipolar stainless steel (0.0045” coated diameter, A-M Systems, Inc) stimulating electrode and a unipolar iridium-coated platinum (0.0080” coated diameter, supplier A-M Systems, Inc) recording electrode were individually lowered into their respective targets under stereotaxic control, with final stimulating electrode coordinates -3.3mm AP, +/-3.0mm ML, -2.6-3.2mm DV (Schaffer collateral) and recording electrode coordinates -3.3mm AP, +/- 2.0mm ML, -2.6-3.2mm DV (CA1 stratum radiatum) (5). Electrode positions within the dorsal-ventral range were optimized to maximize the evoked field excitatory post-synaptic potential (fEPSP). Electrical stimulation of the SC-CA1 pathway was accomplished using computer generated pulses (duration 0.1ms, biphasic) and delivered by a stimulator at low intensities (200µA initially, adjusted later based on response). Evoked field potential responses (fEPSPs) were recorded with a standard differential amplifier and acquisition board, as well as passed through a notch filter, and recorded using computer software WinLTP. The peak amplitude and initial slope of the fEPSP was measured, but slope is reported throughout as it is considered to be a more reliable measure to quantify synaptic strength (6). Baseline evoked responses were then tested at a range of stimulation intensities (0-400µA, in increments of 10µA to 200µA, and then of 20µA to 400µA) to obtain an input-output curve, and a stimulation magnitude evoking 50% of the maximal response was used for the remainder of the recording. Once the actual recording began, evoked field potential responses were sampled at a frequency of 0.033 Hz (1 pulse/30s), with pairs of pulses averaged to give one value per minute (for fEPSP amplitude and slope). In different groups, three stimulation protocols were applied to induce 1) LTD following low-frequency stimulation (LFS, 3Hz, 900 pulses, 5min), 2) weak LTP (wLTP) following one train of high-frequency stimulation (HFS, 100Hz, 1s), or 3) strong LTP (sLTP) following 4 trains of HFS (100Hz, ITI 5min). In drug-free recordings, a stable baseline of at least 15-20minutes was obtained first, before delivery of the LTP/LTD stimulation protocols. The duration of the recording following the induction of LTP/LTD depended on the form of synaptic plasticity as follows: 30min for LTD and wLTP, and 90min for sLTP. In experiments involving drug treatments, saline, ketamine or HNK were injected systemically (ip) 30min, 3.5h or 24h before the induction of sLTP. In the case of the two earlier timepoints, drugs were injected into anesthetized rats following a baseline fEPSP recording (20min), followed by the sLTP protocol 30min or 3.5h later; for the 24h recording, awake animals were pre-treated with drug 24h before conducting the electrophysiological recording the next day (anesthesia, 20min baseline, followed by sLTP protocol 30min later). Animals were euthanized at the end of the recording period by administration of an anesthetic overdose of urethane (4g/kg, ip), without ever regaining consciousness. The primary measure of interest, fEPSP slope (average of two values per minute), was normalized to average baseline fEPSP slope and expressed as a percent (%) of baseline. Data were analyzed by first averaging normalized fEPSP slope (%) into several 5min time bins depending on the recording: LTD (pre-LFS, 5min post-LFS and 30min post-LFS), wLTP (pre-HFS, 5min post-HFS and 30min post-HFS) and sLTP (pre-HFS, 5min post-HFS and 90min post-HFS), as well as an additional pre-drug bin for experiments involving drug treatments. Time bins for normalized fEPSP slope (%) were calculated as follows: pre-drug (average of 5min before drug injection), pre-LFS/HFS (average of 5min before LFS/HFS), 5min post-LFS/HFS (average of 5min after LFS/HFS), 30min/90min post-LFS/HFS (last 5 min of the recording, lasting 30min or 90min after the stimulation protocol), and compared between the different experimental groups.

#### Synthesis and Verification of (2R,6R)-HNK

(2R,6R)-hydroxynorketamine, ((2R,6R)-HNK, (2R,6R)-(-)-2-amino-2-(2-chlorophenyl)-6-hydroxycyclohexanone hydrochloride *aka* (2R,6R)-(-)-hydroxynorketamine hydrochloride) was previously reported and synthesized by Zanos et al. (2016). To a solution of tert-butyl ((1R,3R)-1-(2-chlorophenyl)-3-hydroxy-2-oxocyclohexyl)carbamate (14.3 mmol) in dichloromethane (10 mL) was added trifluoroacetic acid (TFA, 143 mmol, 10 eq.). The reaction was stirred at room temperature for 1 hour. The solvent TFA were then removed by rotary evaporation. The resulting TFA salt was dissolved in water, washed with a 1:1 mixture of saturated aqueous sodium bicarbonate and saturated aqueous potassium carbonate solution, and extracted with ethyl acetate (2X) to give the free base. The ethyl acetate was removed by rotary evaporation. Ethyl acetate (4 mL) was added and HCl in dioxane (4.0 M, 6.0 mL). The suspension was agitated for 30 seconds and then the solid was filtered off and dried under vacuum to give the desired final product (65% yield, total 60 mg) (7).

Chiral HPLC: 98.1% ee (Chiralpak AD column, 60% ethanol in hexanes, 1.0 mL/min), HRMS (ESI+): expected 262.0605 [M+Na] (C12H14ClNO2Na), observed 262.0605 [α]_D_^20^: -92°.

^1^H NMR and ^13^C NMR spectra of the final product were obtained and compared to the known spectra for (2R,6R)-HNK (Figure S1A,B) (7).

### 2. Supplementary Results

#### *Ketamine decreases FST immobility in WKY and WIS rats*

Consistent with the literature, stress-susceptible WKY rats are characterized by dramatic immobility in the forced swim test (FST) compared to normal WIS controls (Figure 1A,B), indicative of abnormal stress coping (average baseline FST immobility for WKY: 258.75 ±6.65s and WIS: 148.33 ±9.28s, non-paired two-tailed t test, p<0.0001). For WKY rats, a 2-way ANOVA (drug treatment x time point) of day2 FST immobility scores indicated only a significant main effect of drug treatment (F1,57=63.05, *p*<0.0001) but no time or drug x time interaction effects (F1,57=0.17, *p=*0.68, n.s. and F1,57=0.29, *p=*0.59, n.s.). Subsequent analyses revealed that ketamine significantly decreased WKY FST immobility compared to that of saline treated WKYs at both time points (30min: from 258.75 ±6.65s to 193.13 ±11.10s; 24h: from 267.14 ±4.56s to 192.00 ±10.71s, Sidak’s *p<*0.0001 for both, Figure 1A). The magnitude of the ketamine effect was comparable at 30min and 24h post-injection (Sidak’s *p>*0.99, n.s.). Importantly, ketamine treatment (5mg/kg, ip) had no effect on general locomotor activity of WKY rats at these time points (n=6/group, 2 way drug x time point ANOVA of OFT total distance travelled revealed only significant main effect of time point (F1,20=6.02, *p=*0.024*),* with no post-hoc pairwise significance, Sidak’s *p>*0.82, n.s., Figure 1C).

For WIS rats (SAL: n=6 and n=6, KET: n=7 and n=7 for 30min and 24h), a 2-way ANOVA (drug treatment x time point) of day2 FST immobility scores indicated significant main effects of drug treatment and time point (F1,22=27.94, *p<*0.0001 and F1,22=6.60, *p=*0.0175) but no significant drug x time interaction (F1,22=0.0018, *p=*0.67, n.s.). Subsequent analyses revealed that ketamine significantly decreased WIS FST immobility compared to that of saline treated WIS rats at both time points (30min: from 148.33 ±9.28s to 96.43 ±8.07s; 24h: from 174.17 ±14.69s to 121.43 ±7.13s, Sidak’s *p=*0.0073 and *p=*0.0063, Figure 1B). The magnitude of the ketamine effect was again comparable at 30min and 24h post-injection (Sidak’s *p=*0.38, n.s.). Importantly, ketamine treatment (5mg/kg, ip) had no effect on general locomotor activity of WIS rats at these time points (n=6/group, 2 way drug x time point ANOVA of OFT total distance travelled revealed no significant effects (F<1.25, *p>*0.28, n.s., Figure 1D)). Therefore, ketamine (5mg/kg, ip) had significant rapid (30min) and sustained (24h) antidepressant effects in both WIS and WKY rats, without affecting general locomotion in either strain.

#### *Stress-prone WKY rats have a significant SC-CA1 LTP deficit*

We were interested in characterizing the WKY model in terms of hippocampal synaptic plasticity, and to this end we performed *in vivo* extracellular recordings at the SC-CA1 synapse in anesthetized rats. Basal evoked fEPSP signals were comparable in WIS (n=42, 21 rats x 2 hemispheres) and WKY (n=38, 19 rats x 2 hemispheres), with similar input-output curves observed in the two strains (Figure 2A). Responses were very similar between the left and right hemispheres in both strains (F<0.08, *p>*0.77, n.s.), so data was combined. A 2-way RM-ANOVA of average absolute fEPSP slope (with strain as the between subject factor and current intensity (0-400µA) as the within subject factor) indicated a significant main effect of current intensity as expected (F25,1950=393.50, *p<*0.0001) but not of strain (F1,78=0.08, *p=*0.78, n.s.), as well as a significant strain x current intensity interaction (F25,1950=4.12, *p<*0.0001). Subsequent analyses revealed no significant differences in average fEPSP between the two strains at any current intensity (Sidak’s *p>*0.66, n.s., Figure 2A), indicating comparable basal synaptic transmission at this synapse. At the highest current intensities (300-400µA), fEPSP slope was consistently slightly lower in WKY compared to WIS rats (maximum fEPSP slope at 400µA: 1.28 ±0.09 vs. 1.46 ±0.09 units/ms, respectively, Figure 2A). Because of the higher WIS maximum as well as the slightly steeper WKY I/O curve slope at this range, the stimulation magnitude evoking ~50% of the maximal response was also slightly higher in WIS compared to WKY rats (~100 vs. 80µA).

Although LTD is generally hard to obtain *in vivo*, we utilized a low-frequency stimulation protocol (3Hz, 900 pulses, 5min) previously reported to have some success, particularly following stress. However, we failed to detect any robust LTD in either WKY or WIS rats (Figure 2B). A 2-way RM-ANOVA of average fEPSP slope (with strain as the between subject factor and time point (pre-LFS, 5min post-LFS and 30min post- LFS) as the within subject factor) indicated a significant main effect of time (F2,42=5.50, *p=*0.0076) but no strain or strain x time interaction effects (F1,21=0.53, *p=*0.48, n.s. and F2,42=0.12, *p=*0.89, n.s.). Subsequent analyses revealed that average fEPSP slope was transiently reduced immediately after the LTD protocol in both strains (5min post-LFS, WKY: 89.34 ±6.18%, n=9, pre-LFS Tukey’s *p=*0.14, n.s.; WIS: 91.78 ±4.35%, n=14, vs. pre-LFS Tukey’s *p=*0.041; 5min post-LFS WIS vs. WKY, Sidak’s *p=* 0.97, n.s., Figure 2B). However, responses fully recovered within 7min in WIS rats (30min post-LFS: 102.45 ±5.89%, vs. pre-LFS Tukey’s *p=*0.99, n.s.), and although fEPSP slope in WKYs remained slightly below baseline levels, near full recovery of responses was observed within the 30min of the recording (30min post-LFS: 96.57 ±6.46%, vs. pre-LFS Tukey’s *p=*0.83, n.s.). Accordingly, average fEPSP slope at 30min post-LFS was not statistically different between the two strains (Sidak’s *p=*0.77, n.s., Figure 2B). Consistent with this, at the end of the recording, a total of 2/14 (14%) of control WIS rats and 1/9 (11%) of WKY rats still expressed robust LTD (defined as 20% or more reduction in fEPSP slope at 30min post-LFS). Therefore, we found no evidence of significant facilitation of LTD in the stress-prone WKY strain compared to normal WIS controls.

Importantly, in contrast to findings on LTD, both weak and strong LTP (wLTP and sLTP, induced by either 1x or 4x trains of HFS: 100Hz, 1s, 5min inter-train interval) at the SC-CA1 synapse were significantly impaired in stress-prone WKYs compared to control WIS rats (Figure 2C-F). In the case of wLTP (Figure 2C,D), a 2-way RM-ANOVA of average fEPSP slope (with strain as the between subject factor and time point (pre-HFS, 5min post-HFS and 30min post-HFS) as the within subject factor) revealed a significant main effects of time and strain (F2,90=39.76, *p<*0.0001 and F1,45=12.36, *p=*0.001), as well as a significant strain x time interaction effect (F2,90=6.58, *p=*0.0022). As expected, average fEPSP slope was significantly increased immediately after the wLTP protocol in both strains (5min post-HFS, WKY: 115.68 ±5.06% of baseline, n=22 and WIS: 134.33 ±4.50% of baseline, n=26, compared to pre-HFS, Tukey’s *p<*0.0001 and *p=*0.0006 for WIS and WKY rats, Figure 2C,D). However, subsequent analyses revealed that this increase was significantly less pronounced in WKY compared to WIS rats (5min post-HFS Sidak’s *p=*0.0007, Figure 2C,D), indicating a deficit in wLTP induction. In addition, despite some decay in both strains (5min post-HFS vs. 30min post-HFS Tukey’s *p=*0.02 and *p=*0.03 for WIS and WKY rats), significant wLTP was still observed at 30min after induction in the control WIS group, whereas post-HFS responses in the WKY strain almost fully recovered within 10min (30min post-HFS WIS: 123.87 ±3.88%, Tukey’s *p<*0.0001; WKY: 104.86 ±3.03%, Tukey’s *p=*0.41, n.s. compared to pre-HFS baseline; 30min post-HFS WIS vs. WKY, Sidak’s *p=*0.0005, Figure 2C,D). Consistent with this, at the end of the recording, responses from 13/26 (50%) control WIS rats still expressed robust wLTP (defined as 20% or more increase in fEPSP slope at 30min post-HFS), with only 2/21 (9.5%) corresponding WKY rats. Therefore, we found strong evidence of deficits of wLTP in induction and/or maintenance at the SC-CA1 synapse of stress-prone WKY rats compared to normal WIS controls.

It is possible that the threshold for LTP induction may be shifted in WKY rats, so that while a weak protocol (wLTP, 1 train of HFS) may not be sufficient, a stronger protocol (sLTP, 4 trains of HFS) could push synapses to express comparable levels of LTP to those in control rats. In the case of sLTP (Figure 2E,F), a 2-way RM-ANOVA of average fEPSP slope (with strain as the between subject factor and time point (pre-HFS, 5min post-HFS and 90min post-HFS) as the within subject factor) revealed a significant main effects of time and strain (F2,48=32.36, *p<*0.0001 and F1,24=7.27, *p=*0.01), as well as a significant strain x time interaction effect (F2,48=4.00, *p=*0.025). Subsequent analyses revealed that, as expected, average fEPSP slope was significantly increased immediately after the sLTP protocol in both strains (5min post-HFS, WKY: 137.90 ±11.32% of baseline, n=12 and WIS: 152.49 ±7.88% of baseline, n=14; vs. pre-HFS, Tukey’s *p<*0.0001 for WIS and WKY rats, Figure 2E,F). Although this increase tended to be less pronounced in WKY compared to WIS rats, differences in sLTP induction between the two strains did not reach statistical significance (5min post-HFS, Sidak’s *p=*0.30, n.s., Figure 2E,F). Therefore, the ability to induce SC-CA1 LTP was mostly rescued in WKY rats by utilizing a stronger LTP protocol. Importantly, however, in control WIS rats, there was no significant decay in potentiation (5min post-HFS vs. 90min post-HFS Tukey’s *p*=0.17, n.s.) and significant sLTP was still observed at 90min after induction (90min post-HFS: 138.40 ±6.00%, vs. 5min pre-HFS Tukey’s *p<*0.0001), whereas post-HFS responses in the WKY group underwent significant decay and almost fully recovered back to baseline within 60-90min (90min post-HFS: 104.44 ±4.91%, vs. 5min pre-HFS Tukey’s *p=*0.76, n.s., vs. 5min post-HFS Tukey’s *p=*0.0006; 90min post-HFS WIS vs. WKY, Sidak’s *p=*0.001, Figure 2E,F). Consistent with this, at the end of the recording, responses from 10/14 (71%) control WIS rats still expressed robust sLTP (defined as 20% or more increase in fEPSP slope at 90min post-HFS), with only 2/12 (17%) corresponding WKY rats. Therefore, while the magnitude and duration of LTP were enhanced by using a stronger induction protocol, we found strong evidence of a significant deficit in the maintenance of sLTP at the SC-CA1 synapse of stress-prone WKY rats compared to normal WIS controls.

#### *Ketamine acutely restores normal SC-CA1 sLTP in WKY rats*

Given the pronounced hippocampal LTP deficit we observed in the WKY model, we aimed to test the effects of ketamine on the impaired sLTP in this strain. Once a stable fEPSP baseline was obtained, saline (1ml/kg) or ketamine (5mg/kg/ml) were administered systemically (ip) to WKY rats and the recording was continued thereafter. Overall, ketamine administration did not have any major effects on SC-CA1 basal synaptic transmission at the 5mg/kg dose in WKY rats (Figure 3A). Next, we tested the effects of ketamine (5mg/kg, ip) on the induction and maintenance of SC-CA1 sLTP in the WKY rat, where the HFS protocol (4 trains of 100Hz, 5min ITI) was delivered at three different time points following ketamine (or saline) administration (30min, 3.5h and 24h). In the case of the early time point (30min post-ketamine, Figure 3A,B), a 2-way RM-ANOVA of average fEPSP slope (with drug as the between subject factor and time point (pre-HFS, 5min post-HFS and 90min post-HFS) as the within subject factor) only revealed a significant main effect of time (F3,45=8.21, *p=*0.0002), but not of drug treatment or the drug x time interaction (F1,15=0.45, *p=*0.51, n.s. and F3,45=0.58, *p=*0.63, n.s.). Subsequent post-hoc analyses indicated there were no significant differences in fEPSP slope between saline (n=12) and ketamine (n=5) treated rats at any time point (Sidak’s *p>*0.51, n.s.). Although fEPSP responses in both groups were potentiated immediately post-HFS (SAL: 137.90 ±11.32% and KET: 122.83 ±11.34% of baseline), this effect was more pronounced, only reaching statistical significance, in the saline group while only a trend was observed in ketamine treated rats, likely due to the lower n (5min pre- vs. post-HFS, SAL: Tukey’s *p<*0.0001, KET: *p=*0.14, n.s., Figure 3A,B). In both groups, however, the observed sLTP was almost completely lost by the end of the recording, as in drug-naïve WKY rats (90min post-HFS SAL:104.44 ±4.91% and KET:107.80 ±6.95%, vs. 5min pre-HFS, Tukey’s *p>*0.76, n.s., with significant decay in the SAL group only: 90min post- vs. 5min post- HFS, Tukey’s *p*=0.0008, Figure 3A,B). Therefore, overall, when the HFS protocol was given 30min after injection, there were no significant effects of ketamine treatment on the SC-CA1 sLTP observed in WKY rats.

Next, we tested the effects of ketamine on SC-CA1 sLTP at 3.5h after injection. Saline and ketamine (3.5h) data from this experiment, as well as saline and (2R,6R)-HNK (3.5h) data from a later experiment were analyzed together in a RM-ANOVA with drug (saline, ketamine or HNK) as the between subject factor and time point as the within subject factor. These data were combined since recordings from saline-treated groups in the two cohorts were not statistically different (F3,126= 0.67, *p=*0.53, n.s.). In the case of the intermediate time point (3.5h post-ketamine, Figure 3C,D), a 2-way RM-ANOVA (drug by time point) of average fEPSP slope revealed significant main effects of time and drug treatment (F3,126=110.2, *p<*0.0001 and F2,42=5.31, *p=*0.0088), as well as a significant drug x time interaction (F6,126=6.53, *p<*0.0001). Subsequent analyses indicated that, as expected, fEPSP responses in both groups were potentiated immediately post-HFS (5min post-HFS, SAL: 159.52 ±9.47% of baseline, n=19; KET: 167.20 ±10.29% of baseline, n=18, vs. pre-HFS, Tukey’s *p<*0.0001 for SAL and KET, Figure 3C,D). Interestingly, regardless of the treatment group, the sLTP induced in WKY rats was more robust when the HFS protocol was given 3.5h compared to 30min after injection (e.g. SAL fEPSP slope at post-HFS: 159.52% vs. 137.90%, respectively; Figures 3C,D and 2E,F), indicating that simply allowing the synapses to recover over a few hours after electrode placement and/or anesthetic administration may facilitate the induction of LTP in this strain. However, while sLTP in saline-treated WKY rats again completely decayed within 90min of its induction as previously observed (90min post-HFS SAL: 102.69 ±4.37% vs. 5min pre-HFS, Tukey’s *p=*0.75, n.s.; vs. 5min post-HFS, Tukey’s *p*<0.0001), robust sLTP was still present at 90min in the ketamine treated group (90min post-HFS, KET: 141.90 ±7.34%, vs. 5min pre-HFS, Tukey’s *p<*0.0001; vs. 5min post-HFS, Tukey’s *p*=0.003, Figure 3C,D). Thus, importantly, while sLTP induction itself was not affected by drug treatment (5min post-HFS Sal vs. KET, Sidak’s *p=*0.99, n.s.), ketamine effectively restored normal SC-CA1 sLTP in WKY rats by significantly facilitating its maintenance (90min post-HFS Sal vs. KET, Sidak’s *p=*0.0003, Figure 3C,D), with the magnitude of synaptic potentiation at 90min in ketamine-treated WKY rats being comparable to that observed in control WIS rats (141.90% and 138.40%, respectively; Figures 3C,D and 2E,F). Consistent with this, at the end of the recording, responses from 13/18 (72%) ketamine-treated WKY rats (vs. 71% of control WIS rats) still expressed robust sLTP (defined as 20% or more increase in fEPSP slope at 90min post-HFS), with only 4/19 (21%) corresponding saline treated WKY rats. Therefore, when the HFS protocol was given 3.5h after injection, ketamine (5mg/kg, ip) completely eliminated the SC-CA1 sLTP deficit in WKY rats, reflecting a robust positive effect on the maintenance of LTP in this strain.

In the case of the late time point (24h post-ketamine, Figure 3E,F), a 2-way RM-ANOVA (drug by time point) of average fEPSP slope only revealed a significant main effect of time (F2,40=17.22, *p<*0.0001), but not of drug treatment or the drug x time interaction (F1,20=0.51, *p=*0.23, n.s. and F2,40=0. 82, *p=*0.45, n.s.). Subsequent post-hoc analyses indicated that there were no significant differences in fEPSP slope between saline (n=12) and ketamine (n=10) treated rats at any time point (Sidak’s *p>*0.22, n.s.). As expected, fEPSP responses in both groups were potentiated immediately post-HFS (5min post-HFS, SAL: 137.90 ±11.32% vs. pre-HFS Tukey’s *p=*0.0004, KET: 143.20 ±12.28% vs. pre-HFS Tukey’s *p=*0.0009, Figure 3E,F). However, while sLTP in saline-treated WKY rats again completely decayed within 90min of its induction (90min post-HFS SAL:104.44 ±4.91% vs. pre-HFS Tukey’s *p=*0.80, n.s., vs. 5min post-HFS Tukey’s *p=*0.0024), only partial decay was observed in the ketamine treated group, so that some sLTP was still present at 90min (90min post-HFS KET: 124.61 ±9.68%, vs. pre-HFS Tukey’s *p=*0.097, n.s., vs. 5min post-HFS Tukey’s *p=*0.17, n.s., 90min post-HFS SAL vs. KET: *p=*0.22, n.s., Figure 3E,F). Consistent with this, at the end of the recording, responses from 5/10 (50%) ketamine treated WKY rats still expressed robust sLTP (fEPSP increase of 20% or more at 90min post-HFS), and only 2/12 (17%) corresponding saline-treated rats. Therefore, when the HFS protocol was given 24h after injection, there was some residual facilitatory effect of ketamine (5mg/kg, ip) on SC-CA1 sLTP in WKY rats.

Unlike experiments where sLTP was induced 30min or 3.5h after injection (i.e. ketamine and saline were administered to anesthetized rats after an input-output curve and a baseline recording were already obtained under drug-free conditions, followed by HFS 30min or 3.5h later); for the 24h time point, awake animals were pre-treated with drug 24h before conducting the electrophysiological recording the next day (since anesthetized rats cannot be kept alive for longer than 6-8h). This unique design of the 24h experiment allowed comparing the input-output curves for saline and ketamine treated WKY rats (SAL: n=12, KET: n=10) at 24h post-injection (Figure 3G). A 2-way RM-ANOVA of average absolute fEPSP slope (with drug treatment as the between subject factor and current intensity (0-400µA) as the within subject factor) indicated a significant main effect of current intensity (F25,1250=174.20, *p<*0.0001, as expected), as well as a significant drug x current intensity interaction (F25,1250=1.92, *p=*0.004), and a trend towards a significant drug treatment effect (F1,50=2.28, *p=*0.14, n.s.). Subsequent analyses revealed no significant differences in average fEPSP between saline and ketamine at any current intensity (Sidak’s *p>*0.31, n.s., Figure 3G). However, average fEPSP slope was consistently higher in ketamine compared to saline treated WKY rats over a wide range of current intensities, where the stimulation magnitude evoking ~50% of the maximal response (i.e. slope of ~0.7) was effectively shifted leftward, from 100µA to 60µA in rats pre-treated with saline compared to ketamine, respectively (Figure 3G). Thus, although its facilitatory effects on sLTP maintenance at 24h were not as pronounced as at 3.5h, ketamine (5mg/kg, ip) additionally caused a dramatic leftward shift of the WKY SC-CA1 input-output curve 24h later, which likely reflects an enhancement of basal synaptic transmission as a result of sustained ketamine-induced potentiation of WKY SC-CA1 synapses at this later time point.

Finally, we tested whether ketamine’s robust facilitatory effect on sLTP under this experimental design is specific to stress-prone WKY rats, which display a pronounced deficit in LTP maintenance. As in WKY rats, ketamine had no major effects on basal synaptic transmission (0-3.5h, Figure 3H) in WIS rats. For sLTP at 3.5h post-ketamine (Figure 3H), a 2-way (drug by time point) RM-ANOVA of average fEPSP slope only revealed a significant main effect of time (F3,21=29.74, *p<*0.0001), but not of drug treatment or the drug x time interaction (F1,7=0.35, *p=*0.57, n.s. and F_3,21_=0.17, *p=*0.92, n.s.). As expected, average fEPSP responses in all WIS rats were potentiated immediately post-HFS (5min post-HFS, SAL: 161.27 ±6.46%, n=3 vs. pre-HFS Tukey’s *p=*0.0003; KET: 168.43 ±12.96%, n=6 vs. pre-HFS Tukey’s *p<*0.0001, Figure 3H). In addition, independent of the treatment group, robust sLTP was still present 90min later (90min post-HFS, SAL: 134.39 ±2.71% vs. pre-HFS Tukey’s *p=*0.028; KET: 136.86 ±12.00% vs. pre-HFS Tukey’s *p=*0.013, Figure 3H). Post-hoc analyses also indicated that there were no significant differences in fEPSP slope between saline and ketamine treated WIS rats at any time point (Sidak’s *p>*0.88, n.s. Figure 3H). Therefore, when the HFS protocol was given 3.5h after injection, ketamine (5mg/kg, ip) had no effect on SC-CA1 sLTP in control WIS rats at this dose.

#### *Ketamine restores long-term spatial memory in WKY rats*

Next, we sought evidence for a functional correlate between SC-CA1 LTP and cognitive deficits on a test of spatial memory mediated by activity in the dorsal hippocampus in the WKY rat. To this end, we first compared performance of WKY and WIS rats on the object location recognition task (NOLRT, Figure 4A), a hippocampal-dependent spatial memory task. Following habituation, rats received training sessions (T1 and T2, 10min each), where two identical objects were placed in two opposing corners of the arena. During the testing session (T3, either 1h or 24h later for short or long -term memory), one of the objects stayed at the familiar location (FL), while the other was moved to a new location (NL) and an NL preference of around/above 60% on the test day indicates good location recognition memory. In the NOLRT task (Figure 4A), 2-way strain x time delay ANOVA of total exploration time during the test session (NL+FL, Figure 4B) indicated only a significant main effect of strain only (F1,35=7.38, *p=*0.010*),* with no post-hoc pairwise significance between the two strains at either time point (Sidak’s *p>*0.77, n.s., Figure 4B). Therefore, although it was slightly higher in WKY rats at both the 1h and 24h delay (1h: 38.00 ±3.19s (n=8) and 42.25 ±3.79s (n=12), 24h: 51.90 ±4.84s (n=10) and 53.66 ±6.24s (n=9) for WIS and WKY, respectively, Figure 4B), total exploration time in the task was comparable between the two strains. A 2-way strain x test delay ANOVA of average test session NL preference (%, NL/NL+FL, Figure 4C) for the drug-free groups (WKY and WIS 1h/24h only) indicated significant main effects of strain and test delay, as well as a significant strain x test delay interaction (F1,35=4.58, *p=*0.039, F1,35=9.02, *p=*0.0049 and F1,35=4.24, *p=*0.047*)*. Subsequent analyses revealed that, as we hypothesized, while short-term location recognition memory was equivalent between the two strains (1h NOLRT NL preference, WKY: 63.74 ±4.22% vs. WIS: 64.09 ±2.16%, Tukey’s *p>*0.99, n.s., Figure 4C), long-term memory was significantly impaired in WKYs (24h NOLRT NL preference, WKY: 41.65 ±6.23% vs. WIS: 59.97 ±3.24%, Tukey’s *p=*0.027; WKY 24h vs. 1h NOLRT NL preference, Tukey’s *p=*0.0037, Figure 4C), consistent with the SC-CA1 LTP deficit in these rats. Consistent with this, one-sample t tests revealed that NOLRT performance (% NL preference) was significantly different from chance (50%) in WIS rats at 1h and 24h (*p=*0.0003 and *p=*0.013) and in WKY rats at 1h (*p=*0.008), indicating significant object recognition memory, while this was not true for WKY rats at 24h (*p=*0.22, n.s., Figure 4C).

Interestingly, although not significantly different from chance (*p>*0.05, n.s. vs. 50%), average NOLRT NL preference at 24h for the WKY strain at baseline was ~41%, instead of being closer to the 50% mark that indicates no discrimination between the objects at the novel and familiar locations and thus, no location recognition memory (i.e. chance performance, Figure 4C). This seems to be due to a small number of WKY rats, which exhibited a selective preference for the object at the FL (e.g. 4/25 SAL WKY rats with NL preference of 0%) and thus skew the group mean away from 50%. These results suggest that while most WKY rats display no significant long-term location recognition memory at a 24h delay (NL preference of around 50%), long-term NOLRT discrimination seems to be intact in a small subset of WKY rats, which in turn exhibit aversion towards novelty, consistent with the neophobia-like phenotype of this strain in other tasks (e.g. open field, novelty-suppressed feeding and social interaction tests).

Next, we tested the effects of ketamine (5mg/kg, ip) on the NOLRT long-term (24h) memory in WKY and WIS rats. Saline or ketamine (WKY: n=25/group and WIS: n=10/group) were injected 3.5h before the second NOLRT training session (T2), i.e. 27.5h before the 24h testing session (Figure 4C, time point was chosen based on our sLTP results). A 2-way ANOVA of average NOLRT NL preference (%) for the 24h test session with strain and drug treatment as the between subject factors indicated a significant main effect of drug treatment *(*F_1,66_=5.03, *p=*0.03*)*, a significant strain x drug treatment interaction *(*F_1,66_=4.39, *p=*0.04*)*, as well as a trend towards a significant main effect of strain *(*F_1,66_=2.16, *p=*0.15, n.s.*)*. Subsequent analyses revealed that long-term object recognition memory at 24h was again impaired in WKY compared to WIS rats (SAL WKY: 41.21 ±4.71% vs. WIS: 58.47 ±3.61%, Tukey’s p=0.05). Importantly, ketamine significantly facilitated WKY long-term object recognition memory at 24h compared to saline-treated WKYs (SAL: 41.21 ±4.71% vs. KET: 62.22 ±3.36%, Tukey’s *p=*0.0008), effectively restoring WKY NOLRT performance to levels comparable to those in control WIS rats at 24h (~60%, Figure 4C), consistent with the positive effects of drug treatment on WKY SC-CA1 sLTP.

Consistent with this, one-sample t tests revealed that while NOLRT performance (% NL preference) at 24h was not significantly different from chance (50%) in saline-treated WKY rats (*p=*0.19, n.s.), significant object recognition memory was observed in ketamine-treated rats (*p=*0.018). In contrast, ketamine (5mg/kg, ip) had no effect on long-term location recognition memory in control WIS rats, as NOLRT NL preference for the 24h test session did not differ between saline and ketamine treated WIS rats (SAL: 58.47 ±3.61% vs. KET: 59.20 ±9.66%, Tukey’s *p=*0.99, n.s., Figure 4C). As in drug-free WIS rats, one-sample t tests revealed significant NOLRT memory compared to chance (50%) (*p<*0.05). Since ketamine increased the WKY average NL preference at 24h to ~60%, as well as effectively eliminating the small subpopulation of WKY rats displaying severe neophobia, it is possible that ketamine’s effects in the task involve a combination of eliminating a tendency toward novelty aversion in a small subset WKY rats and facilitating long-term memory retention in most WKY rats, which present with a spatial memory deficit.

#### *(2R,6R)-HNK restores normal SC-CA1 sLTP and long-term spatial memory without affecting FST immobility in WKY rats*

Given the recent discovery of an active ketamine metabolite and ketamine’s striking effects on WKY SC-CA1 sLTP observed here, we were interested in testing the effects of (2R,6R)-HNK in our model. First, we evaluated (2R,6R)-HNK’s effects on basal synaptic transmission at the SC-CA1 synapse (Figure 5A,B). Once a stable fEPSP baseline was obtained, saline or (2R,6R)-HNK (5mg/kg, ip) were administered to WKY rats (n=19 and n=8, respectively) and the recording was continued for 3.5h thereafter. Although (2R,6R)-HNK increased the variability in fEPSP slope, overall, the metabolite did not have any major effects on SC-CA1 basal synaptic transmission at the 5mg/kg dose in WKY rats (Figure 5A,B).

Next, we directly compared ketamine and its metabolite in terms of their effects on the SC-CA1 sLTP deficit observed in WKY rats, and found that similar to its parent drug, (2R,6R)-HNK had pronounced facilitatory effects at 3.5h post-injection (Figure 5A,B). A 2-way (drug (saline, ketamine from Figure 3C,D or HNK) by time point) RM-ANOVA of average fEPSP slope revealed significant main effects of time and drug treatment (F3,126=110.2, *p<*0.0001 and F2,42=5.31, *p=*0.0088), as well as a significant drug x time interaction (F6,126=6.53, *p<*0.0001, Figure 5A,B). As expected, fEPSP responses in both groups were potentiated immediately post-HFS (5min post-HFS, SAL: 159.52 SAL:102.69% vs. pre-HFS Tukey’s *p=*0.75, n.s.; 90min post-HFS SAL vs. HNK Sidak’s 9.47%, n=19; HNK: 205.14±16.86%, n=8, vs. pre-HFS Tukey’s *p<*0.0001 for SAL and HNK, Figure 5A,B). However, sLTP induction was significantly enhanced in HNK treated rats compared to those that received saline but also ketamine (5min post-HFS HNK vs. SAL, Sidak’s *p=*0.0015, vs. KET, Sidak’s *p=*0.017; Figures 5A,B). In addition, robust sLTP was still present 90min later in the HNK treated group (HNK 90min post-HFS:157.02 ±11.53% vs. pre-HFS, Tukey’s *p<*0.0001), which was similar in magnitude to that following ketamine (90min post-HFS KET vs. HNK Sidak’s *p=*0.93), while no sLTP was observed in the saline group as before (90min post-HFS SAL: 102.69 ±4.37% vs. pre-HFS Tukey’s *p=*0.75, n.s.; 90min post-HFS SAL vs. HNK Sidak’s *p<*0.0001, Figure 5A,B). At the end of the recording, responses from 7/8 (88%) HNK-treated WKY rats still expressed robust sLTP (defined as 20% or more increase in fEPSP slope at 90min post-HFS), compared to 72% for ketamine and 21% for saline (Figure 17C). Despite the slightly higher frequency of sustained sLTP following HNK, average fEPSP slope at 90min post-HSF was not significantly different between HNK and ketamine treated animals (157.02 ±11.53% vs. 141.91 ±7.74%, Sidak’s *p=*0.93, n.s., Figure 5A,B). Therefore, when the HFS protocol was given 3.5h after injection, (2R,6R)-HNK (5mg/kg) also effectively restored SC-CA1 sLTP in WKY rats by significantly facilitating its induction and maintenance, where the magnitude of synaptic potentiation was ~15-20% higher than that observed following an equivalent dose of (R,S)-ketamine (Figure 5A,B).

Next, we tested the effects of (2R,6R)-HNK (5mg/kg, ip) on the NOLRT long-term (24h) memory deficit in WKY rats, as we did for ketamine. Saline (n=25, from Figure 4C), ketamine (n=25, from Figure 4C) or (2R,6R)-HNK (n=15) were injected 3.5h before the second NOLRT training session (T2), i.e. 27.5h before the 24h testing session (Figure 5C, time point was chosen based on our sLTP results). A 1-way ANOVA of average NOLRT NL preference (%) for the 24h test session with drug treatment as the between subject factor indicated a significant effect of drug treatment *(*F=7.75, *p=*0.001*)*. Subsequent analyses revealed that similar to ketamine, (2R, 6R)-HNK significantly restored long-term object recognition memory at 24h in WKY rats (SAL: 41.21 ±4.71% vs. HNK: 61.02 ±5.33%, Tukey’s *p=*0.012; KET vs HNK: *p=*0.98, n.s., Figure 5C) to levels comparable to those in control WIS rats at 24h (~60%, Figure 4C), consistent with the positive effects of drug treatment on SC-CA1 sLTP. Consistent with this, one-sample t tests revealed that while NOLRT performance (% NL preference, Figure 5C) at 24h was not significantly different from chance (50%) in saline-treated WKY rats (*p=*0.19, n.s.), object recognition memory was observed in HNK-treated rats fell just short of significance (*p=*0.058, n.s.).

Next, we tested the effects of (2R,6R)-HNK (5mg/kg, ip) in WKY rats (SAL: n=7/group, HNK: n=9/group for 30min and 24h) in the FST (Figure 5D). A 2-way ANOVA (drug treatment x time point) of day2 FST immobility scores indicated no significant main or interaction effects (F<0.67, *p>*0.42, n.s.). In fact, day2 FST immobility (Figure 5D) was virtually identical between saline and (2R,6R)-HNK treated WKY rats at both 30min (254.29 ±5.71s and 255.00 ±5.20s) and 24h (260.00 ±5.00s and 258.33 ±5.71s) after injection. Therefore, we failed to detect any activity of (2R,6R)-HNK (5mg/kg, ip) in this model using the FST; nor were effects observed on general locomotor activity (n=6/group, 2-way drug x time point ANOVA of OFT total distance travelled indicated no significant effects (F<0.59, *p>*0.45, n.s., Figure 5E).

### 3. Supplementary Figures

**Figure S1A. ^1^H NMR spectrum of (2R,6R)-HNK**


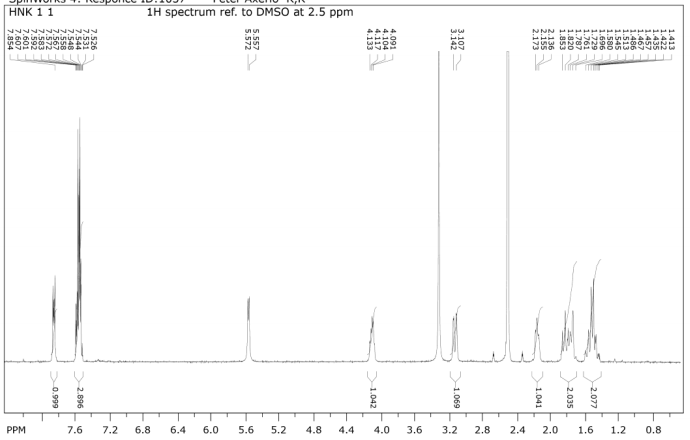


^1^H NMR (400 MHz, DMSO-d6): δ 7.89-7.85 (m, 1H), 7.61-7.53 (m, 3H), 4.10 (dd, J=11.6, 6.7 Hz, 1H), 3.15 (dd, J=14.0, 3.0 Hz, 1H), 2.16 (dddd, J=12.2, 6.6, 4.1, 2.3 Hz, 1H), 1.86-1.70 (m, 2H), 1.59-1.41 (m, 2H) ppm.

**Figure S1B. ^13^C NMR spectrum of (2R,6R)-HNK**


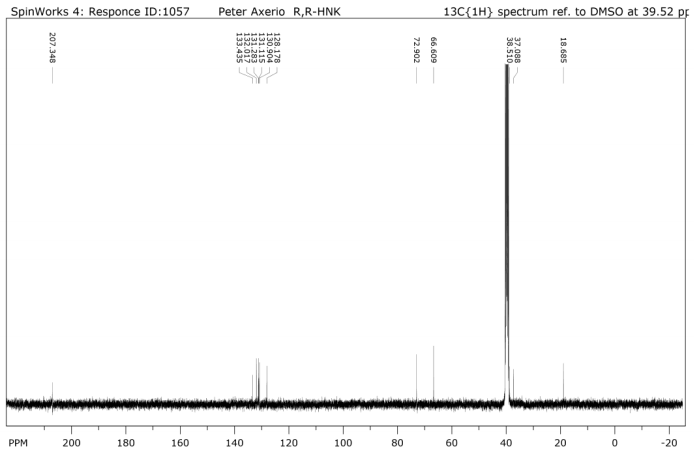


^13^C NMR (100 MHz, DMSO-d6): δ 207.4, 133.4, 132.0, 131.3, 131.1, 130.9, 128.2, 72.9, 66.6, 38.5, 37.1, 18.7 ppm.

### 4. Supplementary References

1. Migues PV, Hardt O, Wu DC, Gamache K, Sacktor TC, Wang YT, Nader K (2010): PKM maintains memories by regulating GluR2-dependent AMPA receptor trafficking. *Nat Neurosci*. 13: 630–634.

2. Vogel-Ciernia A, Wood MA (2014): Examining object location and object recognition memory in mice. *Curr Protoc Neurosci*. 69: 8.31.1-17.

3. Pitsikas N, Boultadakis A, Sakellaridis N (2008): Effects of sub-anesthetic doses of ketamine on rats’ spatial and non-spatial recognition memory. *Neuroscience*. 154: 454–460.

4. Wong TP, Howland JG, Robillard JM, Ge Y, Yu W, Titterness AK, *et al.* (2007): Hippocampal long-term depression mediates acute stress-induced spatial memory retrieval impairment. *PNAS*. 104: 11471–6.

5. Paxinos G, Watson C (2013): *The Rat Brain in Stereotaxic Coordinates : Hard Cover Edition.*, 7th ed. Elsevier Science.

6. Johnston, Wu (1995): *Foundations of cellular neurophysiology*. *MIT Press*. doi: 10.1097/00004691-199703000-00009.

7. Zanos P, Moaddel R, Morris PJ, Georgiou P, Fischell J, Elmer GI, *et al.* (2016): NMDAR inhibition-independent antidepressant actions of ketamine metabolites. *Nature*. 533: 481–6.
